# Genome-wide DNA methylation patterns of bovine blastocysts derived from in vivo embryos subjected to in vitro culture before, during or after embryonic genome activation

**DOI:** 10.1186/s12864-018-4826-3

**Published:** 2018-06-01

**Authors:** Dessie Salilew-Wondim, Mohammed Saeed-Zidane, Michael Hoelker, Samuel Gebremedhn, Mikhaël Poirier, Hari Om Pandey, Ernst Tholen, Christiane Neuhoff, Eva Held, Urban Besenfelder, Vita Havlicek, Franca Rings, Eric Fournier, Dominic Gagné, Marc-André Sirard, Claude Robert, Ahmed Gad, Karl Schellander, Dawit Tesfaye

**Affiliations:** 1 Institute of Animal Science, Animal Breeding and Husbandry Group, University of Bonn, 53115 Bonn, Germany; 2 Institute of Animal Breeding and Genetics, University of Veterinary Medicine Vienna, A-1210 Vienna, Austria; 3 Centre de recherche en biologie de la reproduction, Faculté des sciences de l’agriculture et de l’alimentation, INAF, Pavillon des services, Université Laval, Québec, G1V 0A6 Canada; 4 Department of Animal Production, Faculty of Agriculture, Cairo University, Giza, 12613 Egypt

**Keywords:** DNA methylation, Gene expression, Culture condition, Bovine embryo

## Abstract

**Background:**

Aberrant DNA methylation patterns of genes required for development are common in in vitro produced embryos. In this regard, we previously identified altered DNA methylation patterns of in vivo developed blastocysts from embryos which spent different stages of development in vitro, indicating carryover effects of suboptimal culture conditions on epigenetic signatures of preimplantation embryos. However, epigenetic responses of in vivo originated embryos to suboptimal culture conditions are not fully understood. Therefore, here we investigated DNA methylation patterns of in vivo derived bovine embryos subjected to in vitro culture condition before, during or after major embryonic genome activation (EGA). For this, in vivo produced 2-, 8- and 16-cell stage embryos were cultured in vitro until the blastocyst stage and blastocysts were used for genome-wide DNA methylation analysis.

**Results:**

The 2- and 8-cell flushed embryo groups showed lower blastocyst rates compared to the 16-cell flush group. This was further accompanied by increased numbers of differentially methylated genomic regions (DMRs) in blastocysts of the 2- and 8-cell flush groups compared to the complete in vivo control ones. Moreover, 1623 genomic loci including imprinted genes were hypermethylated in blastocyst of 2-, 8- and 16-cell flushed groups, indicating the presence of genomic regions which are sensitive to the in vitro culture at any stage of embryonic development. Furthermore, hypermethylated genomic loci outnumbered hypomethylated ones in blastocysts of 2- and 16-cell flushed embryo groups, but the opposite occurred in the 8-cell group. Moreover, DMRs which were unique to blastocysts of the 2-cell flushed group and inversely correlated with corresponding mRNA expression levels were involved in plasma membrane lactate transport, amino acid transport and phosphorus metabolic processes, whereas DMRs which were specific to the 8-cell group and inversely correlated with corresponding mRNA expression levels were involved in several biological processes including regulation of fatty acids and steroid biosynthesis processes.

**Conclusion:**

In vivo embryos subjected to in vitro culture before and during major embryonic genome activation (EGA) are prone to changes in DNA methylation marks and exposure of in vivo embryos to in vitro culture during the time of EGA increased hypomethylated genomic loci in blastocysts.

**Electronic supplementary material:**

The online version of this article (10.1186/s12864-018-4826-3) contains supplementary material, which is available to authorized users.

## Background

Following fertilization, the mammalian zygote undergoes successive cleavage divisions in the oviduct and eventually, at the blastocyst stage, the embryo enters to the uterus, hatched from the zona pellucida and attach to the luminal epithelium to initiate implantation [1]. During the early stage of successive developmental processes, the developing embryo undergoes signal communication with the maternal environment [2–4] by secreting signals which subsequently initiates the establishment of persistent bidirectional interaction with the maternal environment [5–7].

Effects of environmental insults on the survival and development of the embryo are highly noticeable at early stages of development when the culture condition induces alterations in genome reprogramming [8, 9] which could even persist until adult life [10]. Several studies indicated the effects of culture condition on the DNA methylation patterns of embryos. For instance, the DNA methylation patterns of key genes such as insulin-induced gene (INSIG) and sterol regulatory element-binding protein (SREBP) were found to be altered in the fetus and placenta in human pregnancies established after in vitro fertilization [11]. Similarly, aberrant hypomethylation of the maternal imprinting control region of the KvDMR1 gene in clinically normal children conceived by in vitro fertilization (IVF) and intracytoplasmic sperm injection (ICSI) was also identified [12]. Moreover, alteration in the global DNA methylation patterns of human placenta derived from pregnancies established from embryos cultured under high oxygen tension [13], and alterations in the allelic methylation patterns of maternally or paternally expressed genes in mice embryonic stem cell fetuses [14] are additional evidences indicating how suboptimal culture condition could affect the genome methylation at the later stage of embryo development. In porcine, the DNA methylation level was increased in in vitro embryos compared to in vivo ones suggesting the adverse effect of in vitro culture on the DNA methylome of embryos [15]. In bovine, oocyte maturation media was confirmed to cause hypermethylation in the liver of fetuses at day 50 of the gestation period [16] indicating the long term effect of culture condition on the DNA methylome of the embryo or the fetal genome.

Previously, we have reported a stage-specific alterations of DNA methylation patterns of in vivo developed blastocysts from embryos developed in vitro up to zygote, 4- or 16-cell stages [17]. In that study, we found that DNA methylation patterns in the resulting blastocysts was compensated in a stage specific manner compared to blastocysts completely developed under in vitro condition. Nevertheless, in that study, it was not possible to predict whether the DNA methylation dysregulation was derived from the epigenetic memory caused by the in vitro oocyte maturation, fertilization and culture condition. Therefore, understanding epigenetic responses of embryos derived from oocytes matured and fertilized in vivo when subjected to in vitro culture conditions is essential to unravel the epigenetic signatures induced by environmental challenges during embryo culture. Therefore, this study aimed to investigate genome-wide DNA methylation patterns of bovine blastocysts derived from in vivo embryos subjected to the in vitro culture condition before, during or after embryonic genome activation.

## Methods

### Animal handling, management and estrus synchronization

Thirty-eight healthy Simmental heifers aged between 15 and 20 months and weighing between 380 and 500 kg in the FrankenForst teaching and research station, University of Bonn, were selected for this study for generation of in vivo embryos. The animals were housed in a free-stall barn with slotted floors and cubicles, lined with rubber mats and they were fed a total mixed ration. Animal handling and management of the experimental animals were performed according to the rules and regulations of the German law of animal protection. The Animal Welfare Committee of the University of Bonn approved the experiment with proposition number 84–02.05.20.12.075.

### In vivo embryo production and in vitro culture

**Fig. 1:**
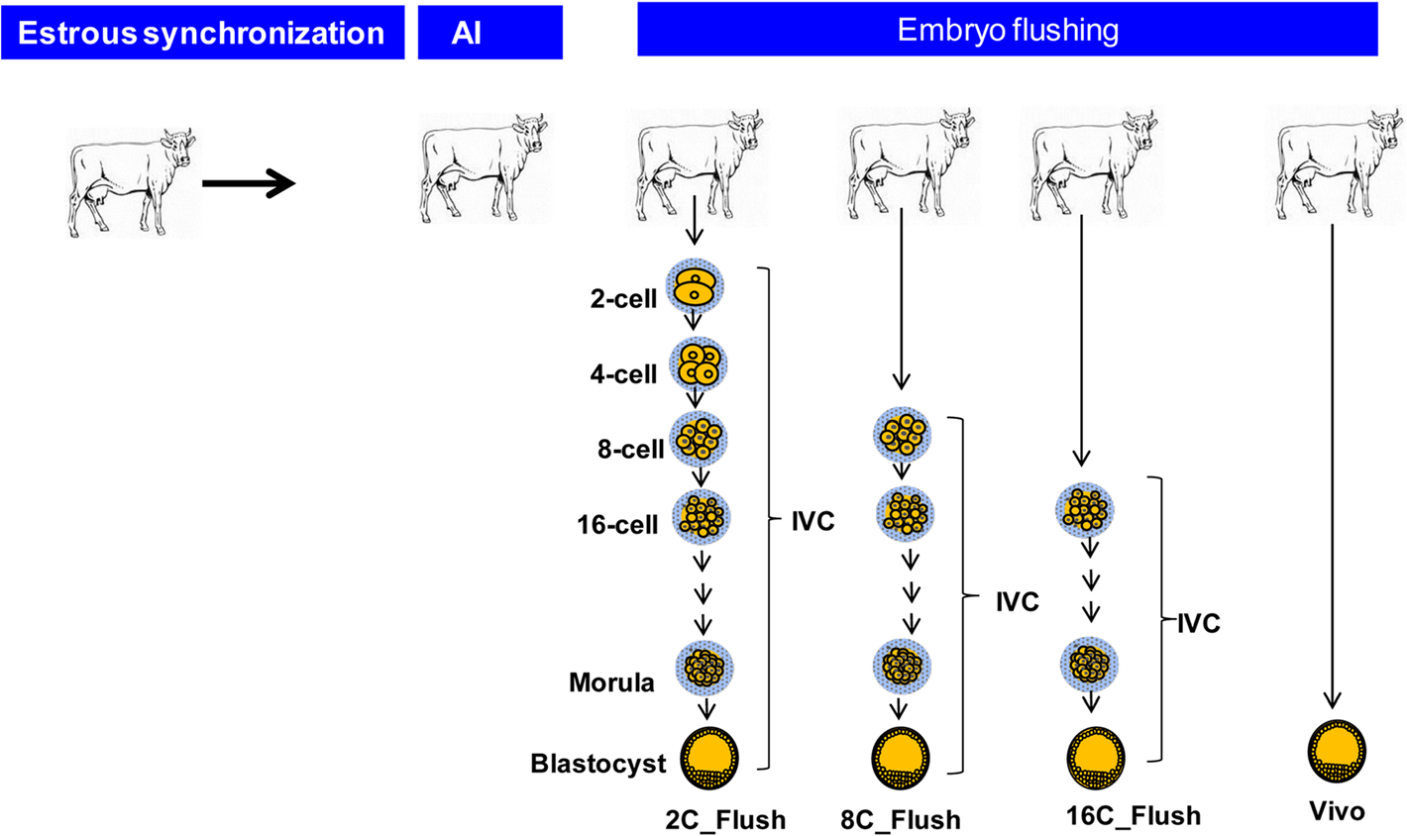
Experimental design used to generate different blastocyst groups for DNA methylation analysis. IVC; In vitro culutre

In this study, different stages of in vivo derived embryos were further cultured in vitro and the resulted blastocysts were used for DNA methylation analysis. For this, Simmental heifers at the FrankenFrost teaching and research station, University of Bonn, were estrous synchronized, super-ovulated and artificially inseminated as described previously [18]. A total of 11, 6, 12 and 13 animals were randomly assigned for 2-, 8–16-cell and blastocyst stage embryo production, respectively. Afterwards, 2-, 8- and 16-cell stage embryos were flushed at 48, 72 and 96 h post insemination, respectively using tubal embryo recovery and transfer methods [19]. The experimental design used for sample collection is indicated in Fig. 1. These embryo groups represent the embryonic developmental stage before, during and after embryonic genome activation, respectively. All embryo groups were then in vitro cultured until the blastocyst stage and the developmental competence of the 2-cell (2C_Flush), 8-cell (8C_Flush) and 16-cell (16C_Flush) were recorded. In addition, the in vivo blastocyst group (vivo) was obtained from embryos completely developed under in vivo condition.

### DNA fragmentation, adapter ligation and methyl sensitive enzymes digestion

Genomic DNA (gDNA) from four replicates (10 blastocysts per replicate) of the 2C_Flush, 8C_Flush, 16C_Flush and vivo blastocyst groups was isolated using the Allprep DNA/RNA micro kit (Qiagen, Germany) following the manufacturer’s recommendations. The gDNA was then fragmented, adapter ligated and cleaved by methyl-sensitive restriction endonucleases using the similar methodology that was used previously ([17]. Briefly, the gDNA of each group was subjected to the MseI enzyme digestion and the MseLig12 (100 μM) (5′-TAA CTA GCA TGC-3′) and 0.5 μl MseLig21 (100 μM) (5-AGT GGG ATT CCG CAT GCT AGT-3′) adapters were ligated to the fragmented DNA using T4 DNA ligase in the presence of 10× One-phor-All buffer PLUS and ATP. Non-methylated genomic regions were then cleaved using FastDigest™ methyl-sensitive restriction endonucleases [HpaII (C/CGG), HinP1I (GC/GC) and Aci1I (C/CGC) (Fermentas, Thermo Fisher Scientific, Germany)]. The efficiency of methyl-sensitive restriction endonucleases cleavage was evaluated based on the qPCR amplification results of the spike-in controls added during the gDNA fragmentation step [17, 20]. Samples which appeared after 5 cycles compared to undigested controls were used for downstream treatments, otherwise the samples were digested one more time using specific methyl sensitive restriction enzyme that did not work in the first run. Afterwards, unmethylated genomic regions were enriched by performing a second round ligation-mediated polymerase chain reaction as indicated previously [20].

### DNA labeling and hybridization

About 2 μg of amplified gDNA from the 2C_Flush, 8C_Flush, 16C_Flush or vivo blastocyst groups was mixed with 1 μl Cy-3 or Cy-5 dyes (Kreatech Biotechnology) and heated at 85 °C for 30 min. After the end of incubation, the samples were purified using the Qiagen PCR purification kit (Qiagen, Germany). Afterwards, 1 μg of Cy5 or Cy3 labeled gDNA sample from the 2C_Flush, 8C_Flush or 16C_Flush and 1 μg Cy5 or Cy3 labeled amplified gDNA of the vivo blastocyst groups were mixed with 157.6 μl hybridization cocktail and incubated at 95 °C for 3 min and at 37 °C for 30 min. At the end of incubation, 65 μl of Agilent-CGHBlock was added and the mix was then transferred onto the EmbryoGENE DNA Methylation Array slides [20]. The slides were then placed in a hybridization oven (Shel Lab) for 40 h at 65 °C at with a speed of 20 RPM. Four biological replicates of 2C_Flush, 8C_Flush and 16C_Flush blastocyst groups were hybridized to four biological replicates of the in vivo group. A total of 12 arrays were hybridized and biological dye-swaps were performed to avoid any false positive results. The arrays were then washed and scanned using PowerScanner (Tecan) which integrates the Array-Pro Analyzer 6.3 software (MediaCybernetics). Agilent’s feature extraction software (Agilent Technologies, USA) was used to extract the array features.

### Array data and downstream analysis

The array data analysis methods used for this study are extensively described previously [17, 20]. Briefly, Loess inter-array normalization method was used to correct the dye effects, while inter-array scale normalization was used to balance the distribution differences among experiments. Linear models for microarray data (limma) [21] was used to identify differentially methylated regions (DMRs). Probes with absolute log_2_ (fold-change) ≥ 1.5 differences between samples to be compared with significant (*p* < 0.05) were considered as differentially methylated regions (DMRs). Thus, probes which showed a significant increase or decrease in signal intensity by ≥1.5 folds in 2C_Flush, 8C_Flush or 16C_Flush compared to the vivo group were considered as hypermethylated and hypomethylated probes, respectively. The pathways enriched by the differentially methylated genes were analyzed using Gprofiler (http://biit.cs.ut.ee/gprofiler/). For comparative analysis of the methylation profile and gene expression data, the genes with absolute log_2_ (fold-change) ≥1.5, *p* value < 0.05 and false discovery rate (FDR) < 0.3 were selected from our previous data (GSE33314 and GSE111990). The heat maps of differentially methylated regions and differentially expressed genes were constructed using PermutMatrix (http://www.atgc-montpellier.fr/permutmatrix/) and matrix2png interface (https://matrix2png.msl.ubc.ca/bin/matrix2png.cgi). The network of genes was visualized using the networkanalyst tool (http://www.networkanalyst.ca/).

### Confirmation of differentially methylated genomic regions using bisulfite sequencing

The DNA methylation levels of selected candidate genes were validated using bisulfite sequencing. For this, the sequences of the candidate gene/probe were retrieved using the Site Search tool http://emb-bioinfo.fsaa.ulaval.ca/bioinfo/html/SiteSearch.html. Afterwards, gene/sequence specific primers (Additional file 1: Table S1) were designed using MethPrimer (http://www.urogene.org/cgi-bin/methprimer/methprimer.cgi) and used for amplification of the gene of interest. To do this, first the genomic DNA of each blastocyst group was modified with sodium bisulfite converted using EZ DNA methylation direct kit (ZymoResearch). Afterwards, bisulfite converted gDNA was subjected to PCR amplification using Zymo Taq™ DNA polymerase (ZymoResearch). The PCR product was purified and ligated to the pGEM®-T Easy Vector System (Promega, USA) and transformed to *E. coli* competent cells. The bacterial culture was plated onto LB agar/ampicillin/IPTG/X-gal plate and incubated overnight at 37 °C. After overnight culture independent white colonies were selected and sequenced with GenomeLab™ GeXP Genetic Analysis System (Beckman Coulter). The bisulfite sequencing data were then analyzed using the quantification tool for methylation analysis (http://quma.cdb.riken.jp/).

### Quantification of candidate genes using quantitative real time PCR

Since the DNA methyltion profile data was supperimposed to previously generated expression data [[22], GSE33314 and GSE111990]] using the same experimental design, expression patterns of selected differentially expressed genes were further quantified using quantitative polymerase chain reaction (qPCR) using gene specfic primers (Additional file 2: Table S2) designed using primer3 (http://bioinfo.ut.ee/primer3-0.4.0/). For this, the total RNA isolated from the experimental blastocyst groups were reverse transcribed. The qPCR was then performed in 20 μl reaction volume containing iTaq SYBR Green Supermix with ROX (Bio-Rad laboratories, Germany), the cDNA samples of each treatment group and the specific forward and reverse primer in the StepOnePlus™ Real-Time PCR Systems (Applied Biosystems, USA). The qPCR thermal cycling parameters were set to 95 °C for 3 min followed by 40 cycles of 95 °C for 15 s and 60 °C for 1 min. Following this, dissociation curve was generated by starting fluorescence acquisition at 60 °C and measurements were taken in every 7 s interval until the temperature reached 95 °C. The specificity of amplification was evaluated by monitoring the dissociation (melting) curve of each candidate gene. The qPCR data was generated from three independent biological replicates and the data was analyzed using the delta Ct method (2^−ΔΔCt^). The expression level of Glyceraldehyde 3-phosphate dehydrogenase (GAPDH) was used as an endogenous normalizer.

## Results

### DNA methylation marks sensitive to in vitro culture condition at any stage of embryonic development

**Fig. 2:**
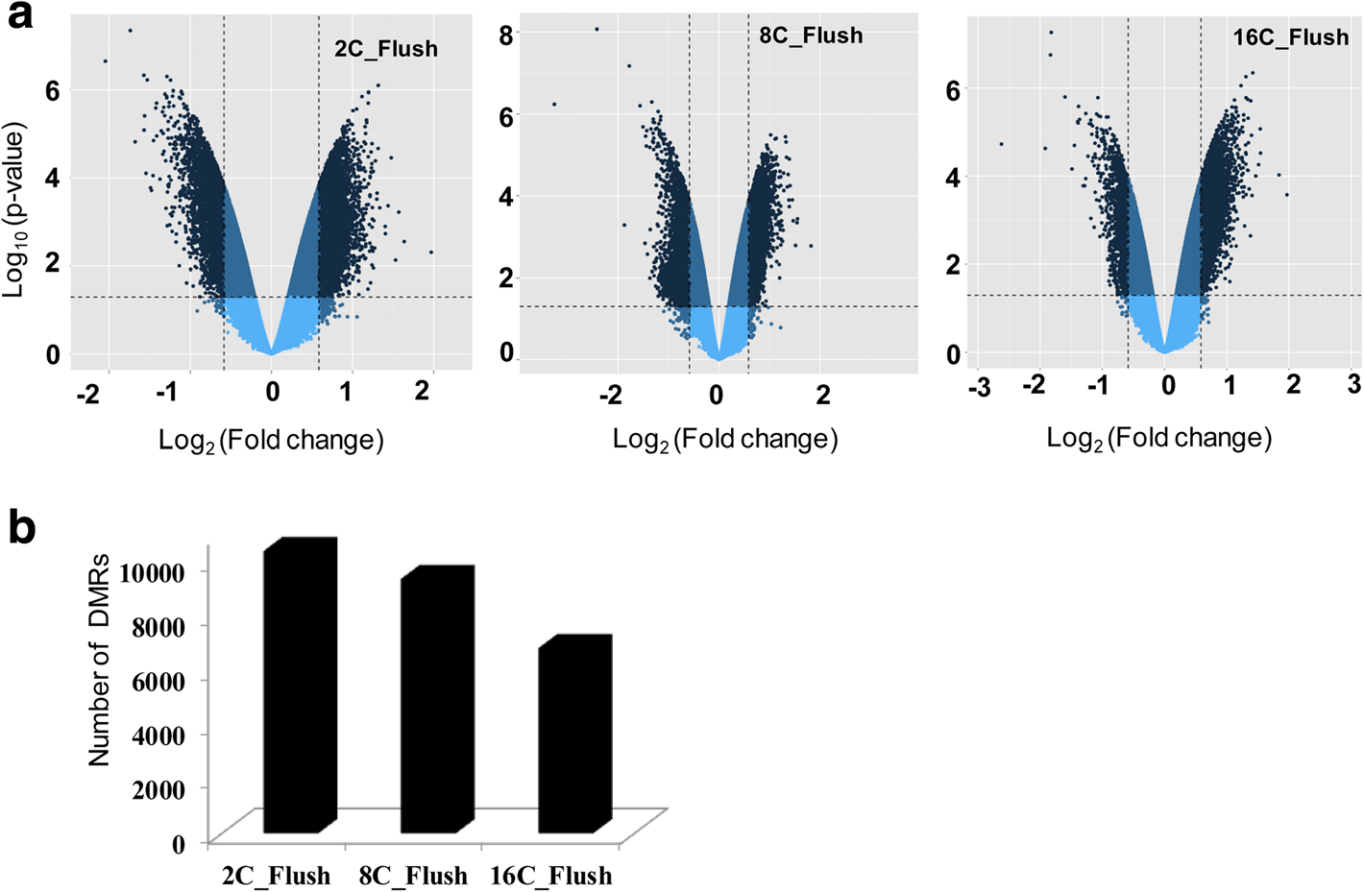
Global DNA methylation patterns of differentially methylated genomic regions in different blastocyst groups. **a** Volcano plots displaying the distribution of hypermethylated (positive log_2_ fold changes) and hypomethylated (negative log_2_ fold changes) genomic loci in each blastocysts group. **b** The total number of differentially methylated genomic regions in each blastocyst group

In this study, the DNA methylation patterns of blastocysts derived from embryos developed in vivo followed by in vitro culture were investigated. For this, in vivo developed 2-cell (2C_Flush), 8-cell (8C_Flush) and 16-cell (16C_Flush) embryos were subjected to in vitro culture condition until the blastocyst stage. Although the day 9 blastocyst rate was similar in the 2C_Flush and 8C_Flush groups, the day 7 and day 8 blastocyst rates were relatively lower in 2C_Flush embryos. The day 7–9 blastocyst rates in the 16C_Flush group were relatively higher compared to both the 2C_Flush and 8C_Flush groups (Table 1). Therefore, to get an overview of the epigenome reprogramming that occurred in these embryo groups, we investigated DNA methylation patterns in each of the blastocysts group using EmbryoGENE DNA methylation array platform. Aberrant hyper or hypomethylated genomic loci were increased in blastocysts derived from embryos that were exposed to in vitro conditions at 2- and 8-cell stages compared to the 16-cell stage indicating that the suboptimal culture condition induced aberrant DNA methylation pattern more strongly before and during the embryonic genome activation than later (Fig. 2).

**Table 1:** Developmental competence of in vivo developed 2-, 8- and 16-cell stage embryos under in vitro culture condition

Group	Heifers	Total embryo flushed	Blastocyst development					
			Day 7		Day 8		Day 9	
	(n)	(n)	(n)	(%)	(n)	(%)	(n)	(%)
2-cell flush	11	69	15	21.7^a^	42	60.9 ^a^	57	82.6^a^
8-cell flush	6	118	63	53.4^b^	93	78.8 ^b^	97	82.2^a^
16-cell flush	12	45	36	80.0^c^	42	93.3 ^c^	45	100.0^b^

**Fig. 3:**
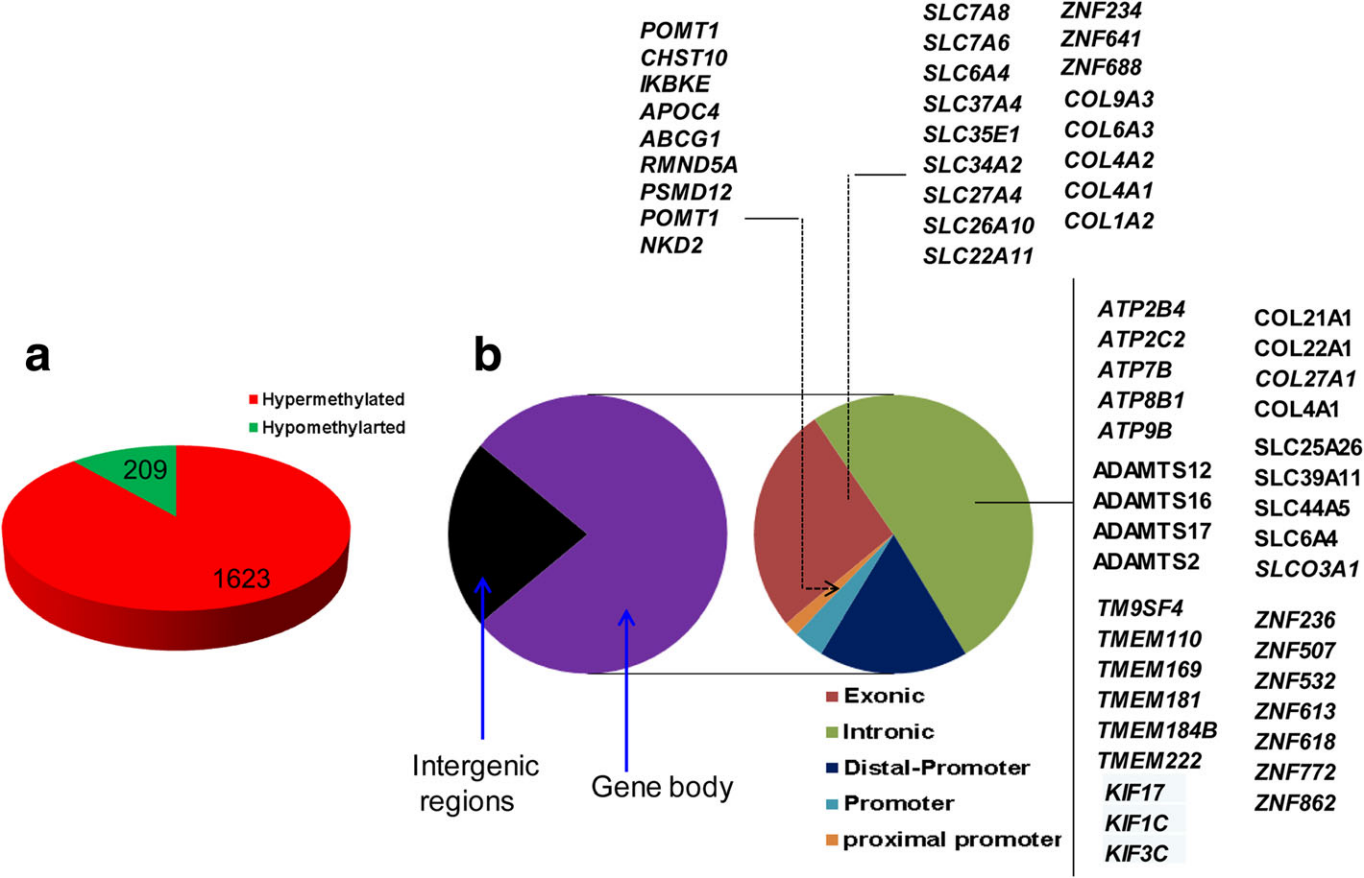
Commonly differentially methylated genomic regions in all blastocyst groups. **a** The proportion of commonly detected hypermethylated and hypomethylated genomic loci. **b** Genomic localization of commonly differentially methylated genomic regions and the list of representative genes in each category

**Fig. 4:**
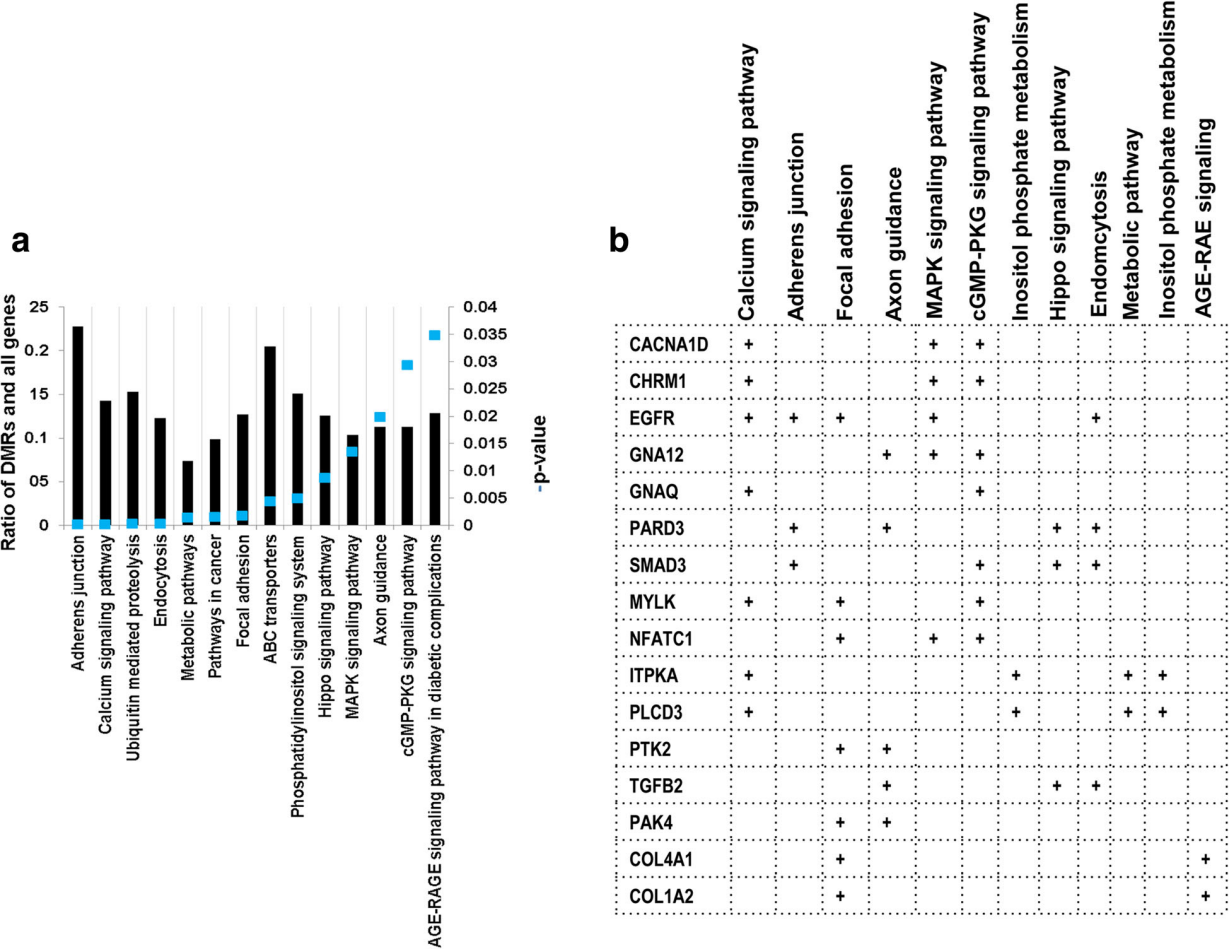
**a** List of pathways enriched by commonly differentially methylated genomic regions in all blastocyst groups and **b** list of representative differentially methylated genomic regions involved in more than one pathway

A detailed analysis of DMRs including 160 CpG islands indicated that the DNA methylation patterns of 1832 genomic loci (containing 29,166 CpG sites) were differentially methylated in all the three blastocyst groups, despite the stage specific exposure to the in vitro culture (Fig. 3a). These genomic loci represented 17.6, 19.6 and 26.7% of the total DMRs identified in the 2C_Flush, 8C_Flush and 16C_Flush, respectively. Among these, 88.5% (*n* = 1623) of the DMRs including the imprinted genes (*PEG3, IGF2R, ASB4, SFMBT2* and *GRB10*) and *DNMT1* were found to be hypermethylated. The majority of commonly altered genes including the solute carrier clusters, collagens, ADAM metallopeptidases, ATPases, zink finger proteins, kinesins were differentially methylated in the exonic and/or intronic gene body regions (Fig. 3b). These differentially methylated genes were found to be involved in 16 biological pathways including the calcium signaling pathway and focal adhesion (Fig. 4a). In addition, *PAK4, COL4A1, COL1A2, EGFR, SMAD3, ITPKA* and *MYLK* were among the commonly differentially methylated genes that were found to be involved in two or more pathways (Fig. 4b).

**Fig. 5:**
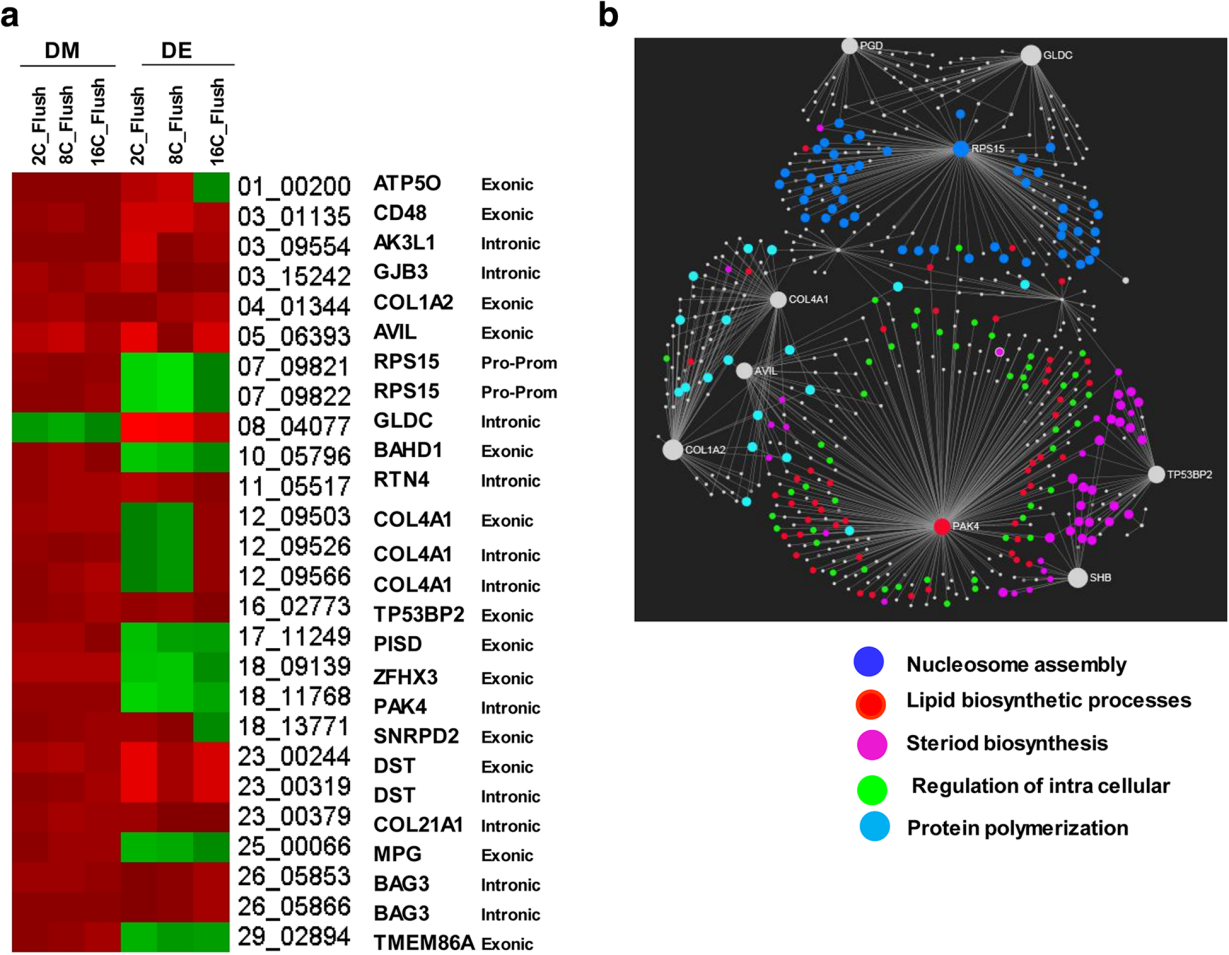
**a** The heatmap depicting the methylation and expression patterns of DMRs commonly detected in all blastocyst groups. DM; differentially methylated, DE; differentially expressed. The red color indicates hypermethylation of the DNA methylation or upregulation of the mRNA expression whereas the green color indicates hypomethylation of the DNA methylation or downregulation of the mRNA expression. **b** The gene network and biological processes enriched by commonly detected DMRs and which showed inverse correlation with their corresponding mRNA expression levels

After identification of genomic regions commonly differentially methylated in all blastocyst groups, we investigated the effects of aberrant DNA methylation patterns on the blastocyst gene expression by superimposing the DNA methylation profile with the transcriptome data [[22]], GSE33314 and GSE111990]]. The results of this analysis indicated that only 2% (*n* = 28) of the annotated differentially methylated genes were differentially expressed in all three groups. Among which, the expression level of 11 genes including *BAHD1, RPS15, PAK4* and *PISD* were negatively correlated with the DNA methylation patterns (Fig. 5a). Network analysis of these genes indicated that *PAK4* and RPS15 genes which were downregulated but hypermethylated in all blastocysts group were found to be the center genes in lipid biosynthesis and nucleosome assembly, respectively while TP53BP2 was the center gene of inflammatory response and intracellular transport pathways (Fig. 5b).

### Aberrant DNA methylation patterns induced in both blastocysts developed in vitro from embryos flushed before and during embryonic genomic activation

**Fig. 6:**
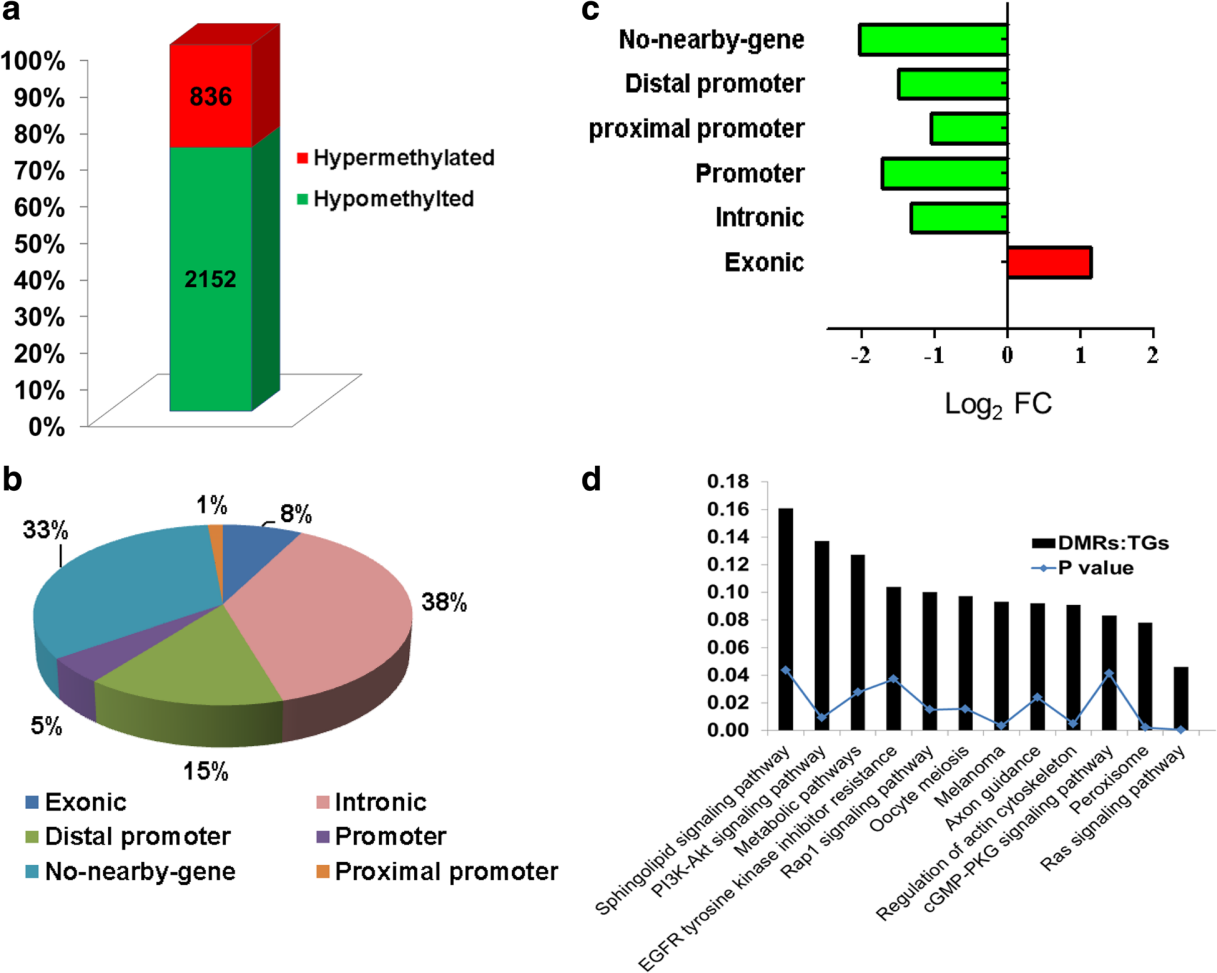
Commonly differentially methylated genomic regions in both the 2C_Flush and 8C_Flush blastocyst groups. **a** The proportion of hypermethylated and hypomethylated DMRs. **b** The distribution of DMRs in different genomic regions. **c** The proportion of hypermethylated and hypomethylated DMRs in different genomic regions. Log_2_ FC: the ratio of hypermethylated and hypomethylated genomic loci described in Log_2_ scale. **d** Pathways enriched by differentially methylated genomic regions in both the 2C_Flush and 8C_Flush blastocyst groups. DMRs:TGS, the ratio of differentially methylated genomic regions (DMRs) and the total number of genes (TGs) involved in pathway

Unlike the 16C_Flush blastocysts group, both the 2C_Flush and 8C_Flush blastocysts were obtained from embryos that spent several cleavages in vitro including the major embryonic genome activation (EGA). Therefore, here we investigated the DMRs commonly dysregulated only in the 2C_Flush and 8C_Flush groups, but not in the 16C_Flush group. The results showed that including DNA (cytosine-5)-methyltransferase 3 beta (DNMT3B), the methylation patterns of 2988 genomic loci were altered only in these two blastocyst groups of which 72% were hypomethylated in both blastocyst groups (Fig. 6a). Genomic localization of these DMRs showed that about 67% of the DMRs are localized in and around the gene body regions (Fig. 6b) and the hypermethylated loci were more common in the exonic regions and the hypomethylated loci were common in the promoter and intronic regions (Fig. 6c).

Next, we investigated the function of these genomic regions by performing gene enrichment analysis and the results have shown that Pi3-Akt signaling pathway, Ras signaling pathway, peroxisome pathway, EGFR tyrosine kinase inhibitor resistance and metabolic pathways were among the biological pathways altered by the differentially methylated genes (Fig. 6d).

**Fig. 7:**
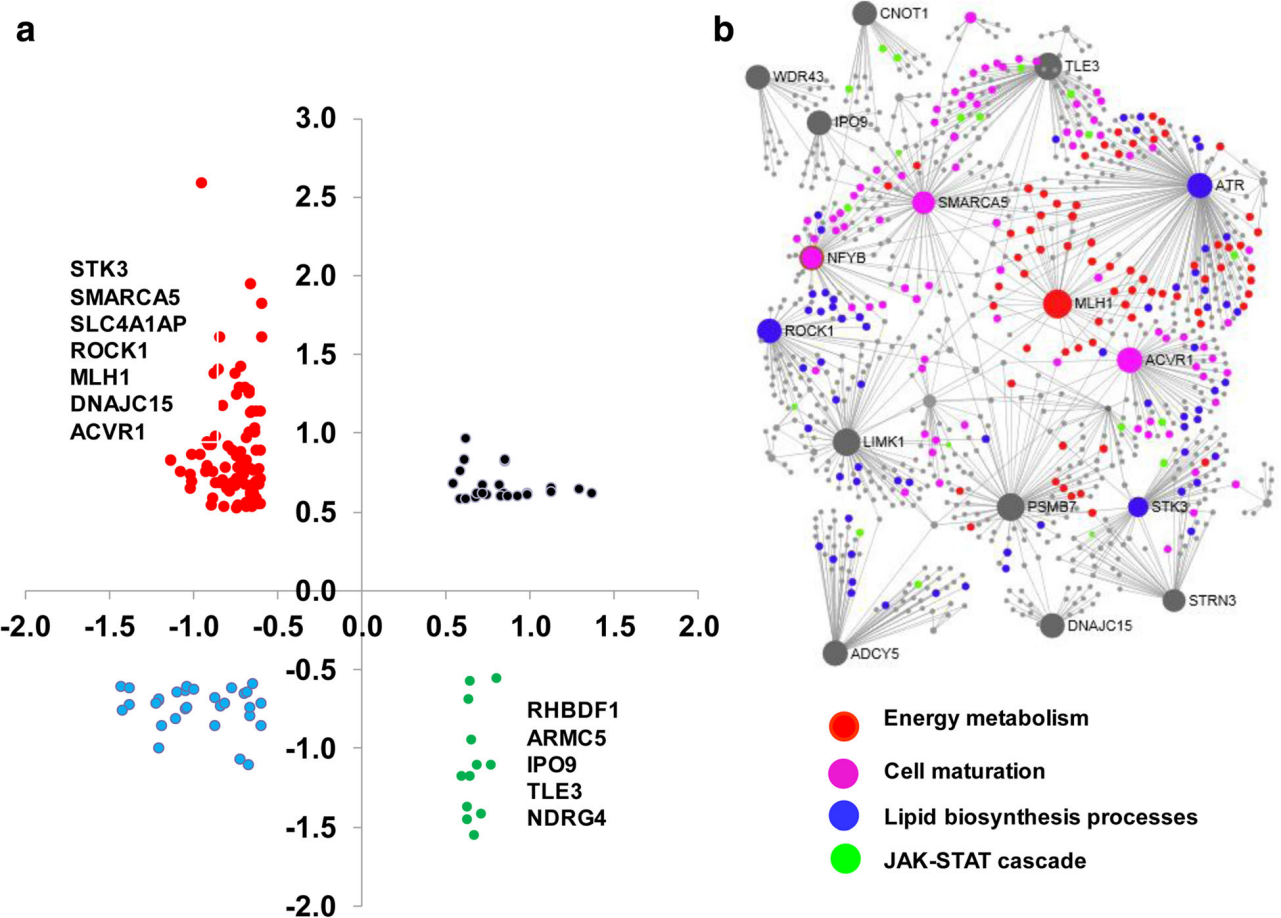
**a** The scatter plot displaying expression patterns and the methylation levels of DMRs commonly detected in the 2C_Flush and 8C_Flush groups in blastocysts of the 2C_Flush. The DNA methylation and mRNA expression levels are indicated in the X and Y axis, respectively. **b** The gene network and biological processes enriched by these DMRs whose expression levels arenegatively correlated with mRNA expression levels

Comparative analysis of the methylation and mRNA expression patterns indicated that the methylation levels of 72 out of 753 annotated DMRs were inversely correlated to their corresponding expression levels. Among these, the expression level of 61 hypomethylated genes including *MLH1*, *SMARCA5, NFYB, ATR, ROCK1* and *ACVR1* was upregulated while the mRNA level of 11 hypermethylated genes including *ADCY5* and IGF2R was downregulated in both 2C_Flush and 8C_Flush blastocyst groups (Fig. 7a). Furthermore, the gene network analysis showed that *SMARCA5, NFYB* and *ACVR1* were the center genes regulating cell maturation and *ATR, ROCK1* and *ADCY5* were found to be the center genes of the lipid biosynthesis processes (Fig. 7b).

### DNA methylation patterns induced exclusively in blastocysts developed in vitro from embryos flushed before, during or after embryonic genomic activation

**Fig. 8:**
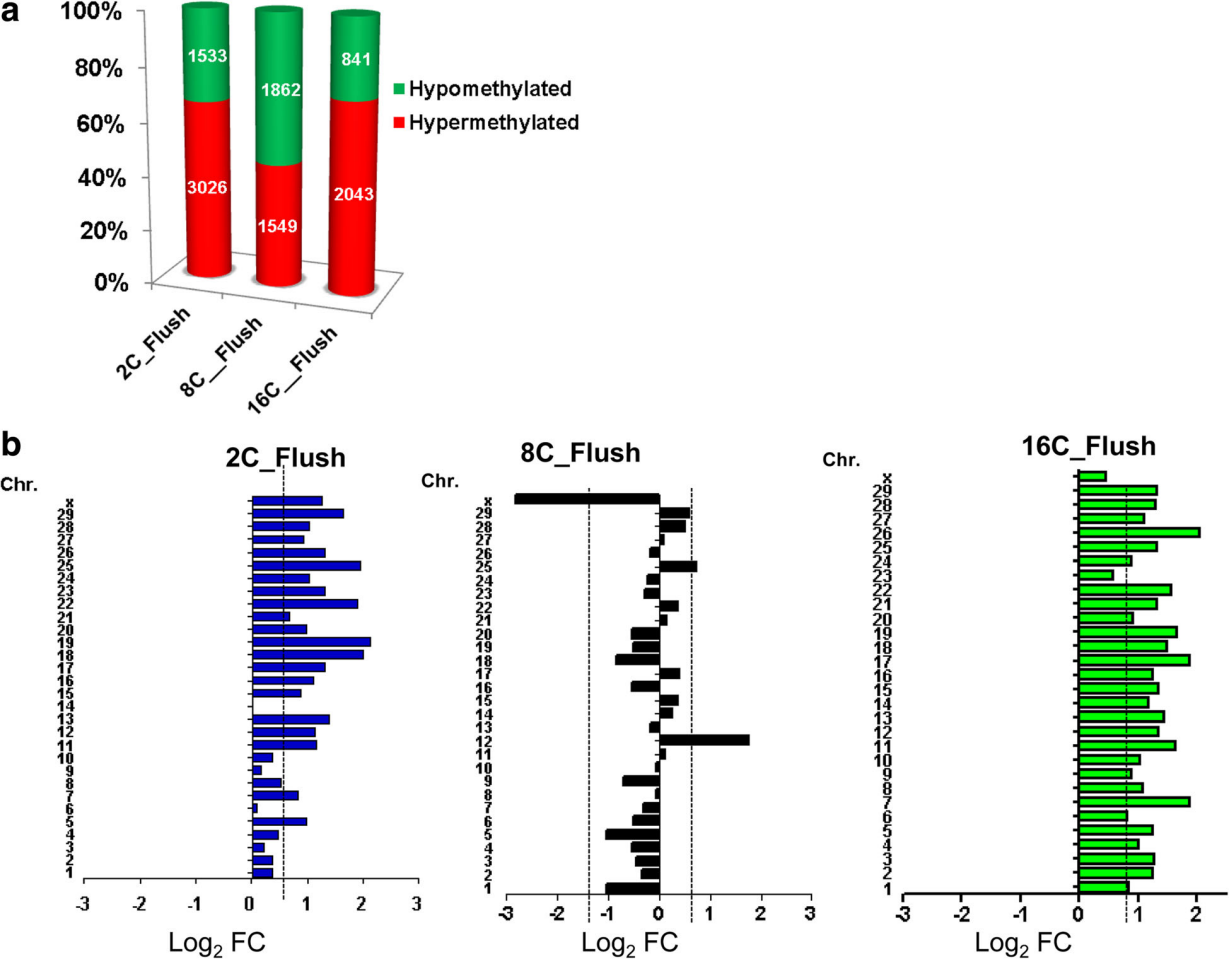
**a** the number and **b** chromosomal enrichment of genomic loci differentially methylated exclusively in 2C_Flush, 8C_Flush or 16C_Flush blastocyst group. Chr, Chromosome, Log_2_ FC: the ratio of hypermethylated and hypomethylated genomic loci described in Log_2_ scale

We considered that in the 2C_Flush blastocysts group, both the major and minor EGA occurred under the in vitro condition, while in the 8C_Flush blastocyst group, minor EGA was completed under in vivo condition whereas the major EGA was partly occurred under in vivo condition. In the 16C_Flush blastocyst group, both minor and major EGA was completed under in vivo condition. Therefore, we further analyzed the differentially methylated genomic regions that were specific to the 2C_Flush, 8C_Flush or 16C_Flush blastocyst groups. Our findings revealed that a total of 4599 DMRs including kruppel-like factors (KIF1, − 4, − 6, − 7, − 8, − 11, − 16, and KLF22) were unique to the 2C_Flush blastocysts whereas a total of 3410 DMRs including ankyrin repeat domain-containing protein (*ANKRD6*,-16, − 24, − 28, − 35, − 39 and ANKRD60) were unique to the 8C_Flush group. Conversely, a total of 2889 DMRs including elongation factors (*EIF2AK1*, *EIF3C* and *EIF4B*) were found to be unique to the 16C_Flush blastocysts group. When these genomic loci were partitioned into hypermethylated and hypomethylated categories, the hypermethylated loci exceeded the number of hypomethylated genomic loci in the 2C_Flush and 16C_Flush groups. However, the reverse was observed in the 8C_Flush group (Fig. 8a).

### Chromosomal distribution and genomic localization of exclusively differentially methylated loci in blastocysts developed in vitro from embryos flushed before, during or after embryonic genomic activation

To further identify chromosomal regions sensitive to the in vitro culture condition, we looked into the chromosomal distribution of the DMRs induced exclusively before, during or after EGA. To perform this, we first arranged the chromosome numbers based on the total probe and DMR densities (Additional file 3: Figure S1). The frequency of the DMRs in the chromosomes tended to follow the probe density available in the EDMA with minor deviations. However, the number of differentially methylated genomic loci at X chromosome was 1.8 and 2.6 times higher in the 2C_Flush compared to the 8C_Flush and 16C_Flush groups, respectively. This has been also evidenced in the circular circos plot which shows the distribution of significantly differentially methylated genomic loci across the chromosomes (Additional file 4: Figure S3). In addition, chromosomal enrichment analysis (analysis of hypermethylated probes with reference to hypomethylated probes) of the DMRs revealed that unlike the 8C_Flush group, the proportion of hypermethylated loci exceeded the hypomethylated ones in all chromosomes in the 2C_Flush and 16C_Flush groups. However, in the 8C_Flush, unlike other groups, four times higher hypomethylated probes than hypermethylated loci were detected on X chromosome (Fig. 8b).

**Fig. 9:**
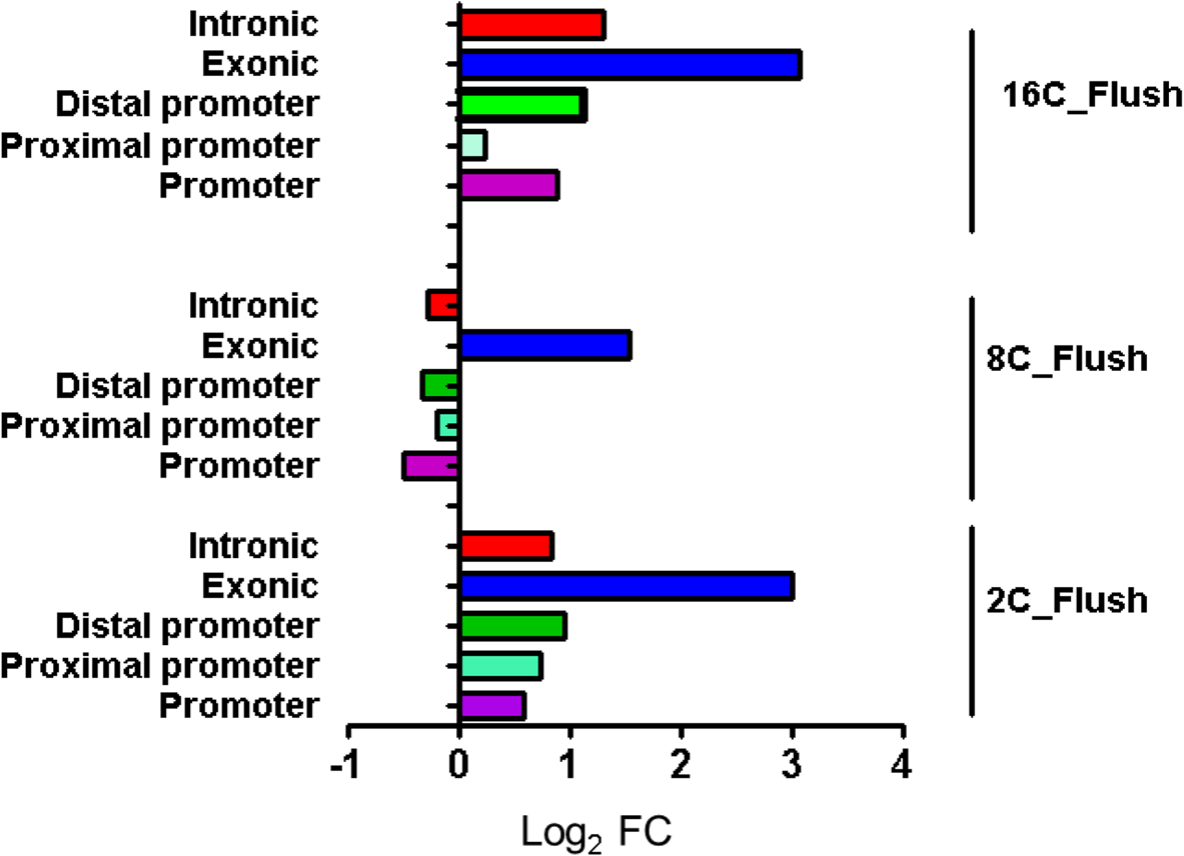
Genomic enrichment of the DMRs which are unique to each blastocyst group. Log_2_FC: the ratio of hypermethylated and hypomethylated genomic regions described in Log_2_ scale

Uniquely differentially methylated genomic loci were subjected to the genomic localization and genomic enrichment analysis. The results indicated that 72.7, 71.4 and 74.2% of the DMRs in the 2C_Flush, 8C_Flush and 16C_Flush blastocysts, respectively were located in and around the gene body regions, whereas the rest were localized in the intergenic regions (Additional file 5: Figure S3). Within the gene body regions, higher proportions of unique DMRs were located in the intronic followed by exonic regions whereas the proportion of DMRs on the plain and proximal promoter was relatively lower in all blastocyst groups. However, the hypermethylated genomic loci exceeded the hypomethylated ones in the exonic regions of all blastocyst groups and the hypermethylated genomic loci surpassed the hypomethylated ones in the plain promoter, proximal promoter, distal promoter and intronic regions of the 2C_Flush and 16C_Flush blastocyst groups (Fig. 9).

**Fig. 10:**
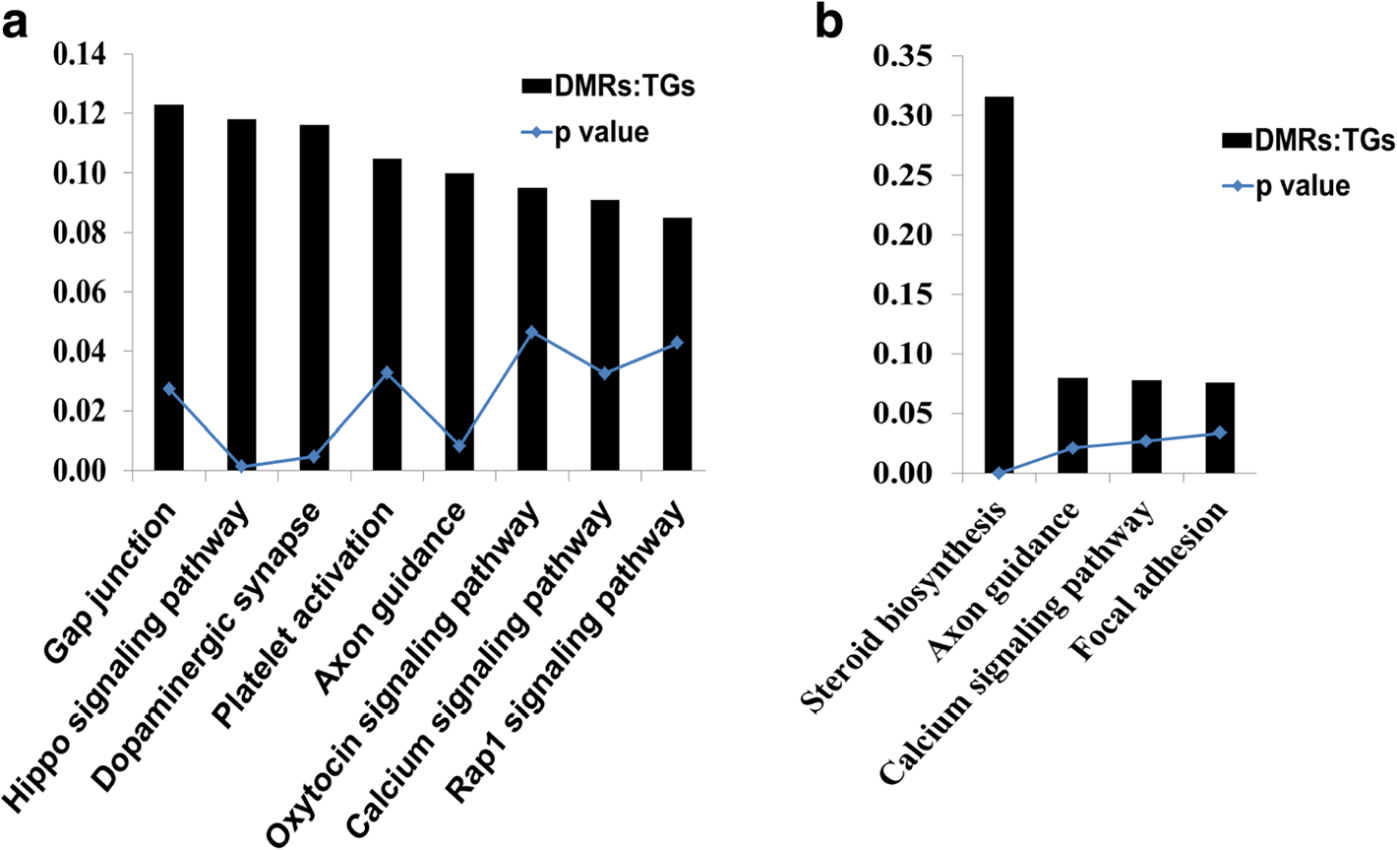
Pathways enriched by the DMRs exclusively detected only in the 2C_Flush (**a**) and 8C_Flush (**b**) blastocyst groups. DMRs:TGS, the ration of differentially methylated genomic regions (DMRs) and the total number of genes (TGs) involved in the pathway

In order to investigate the biological relevance of the DMRs exclusively induced in blastocysts derived from embryos subjected to the in vitro culture condition before, during or after major EGA, annotated DMRs were subjected to pathway analysis. Results showed that differentially methylated genes only in the 2C_Flush were significantly enriched in 15 biological pathways including Hippo signaling pathway. On the other hand, the genes differentially methylated only in the 8C_Flush group were significantly enriched in steroid biosynthesis, axon guidance, calcium signaling and focal adhesion pathways (Fig. 10). However, exclusively differentially methylated genes in the 16C_Flush group didn’t display any significant enrichment in any of the biological pathways.

### The expression patterns of exclusively differentially methylated genomic loci in blastocysts developed in vitro from embryos flushed before, during or after embryonic genomic activation

**Fig. 11:**
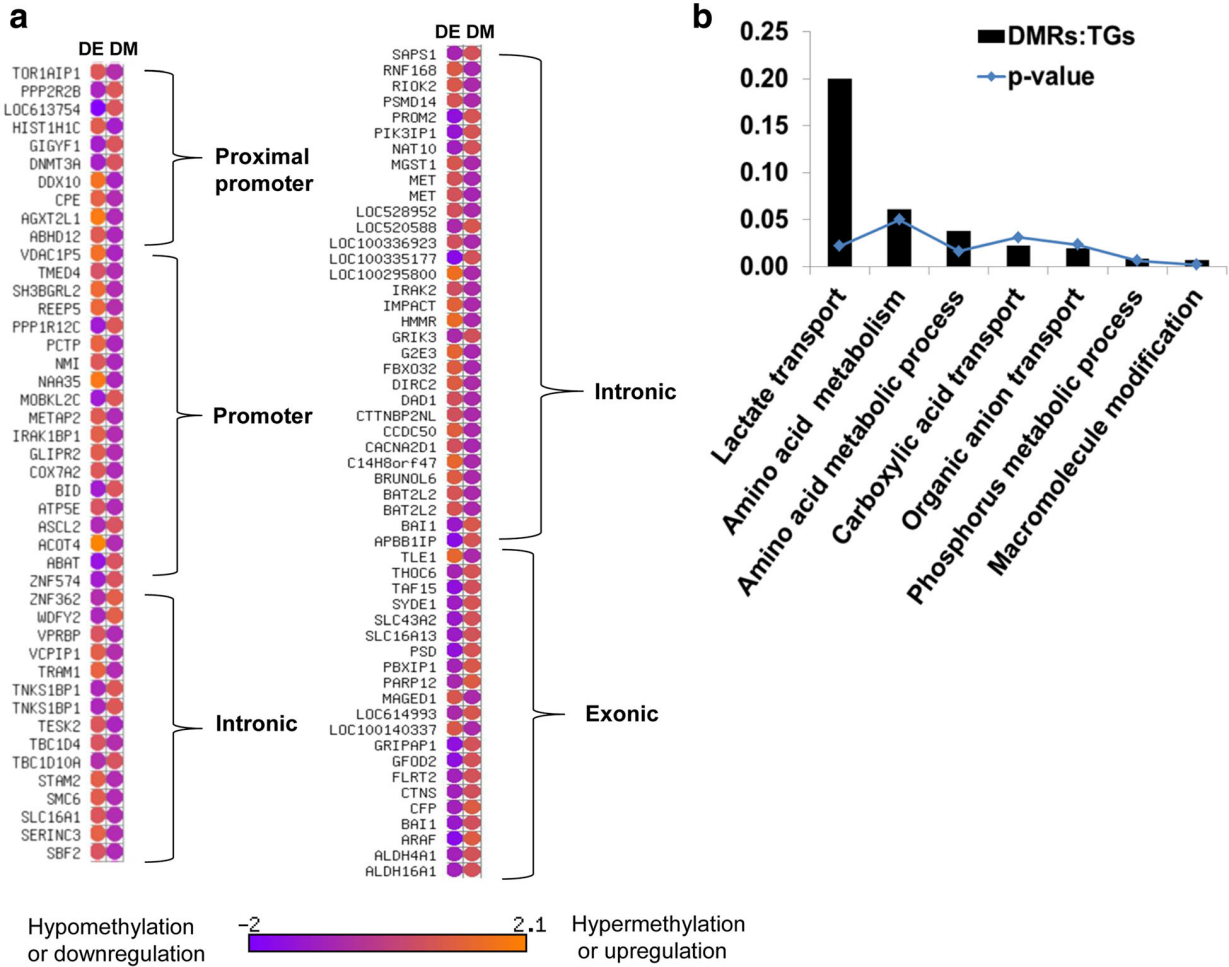
**a** The expression patterns of the DMRs exclusively detected in the 2C_Flush blastocysts group. DM; differentially methylated, DE, differentially expressed. **b** The pathways enriched by DMRs whose DNA methylation patterns were inversely correlated with the mRNA expression levels. DMRs:TGS, the ration of differentially methylated genomic regions (DMRs) and the total number of genes (TGs) involved in the pathway

Comparative analysis of DNA methylation and gene expression showed that a total of 179 exclusively differentially methylated genes in 2C_Flush were mapped to 167 differentially expressed genes and the methylation patterns of 94 DMRs were inversely correlated with their corresponding mRNA expression patterns (Fig. 11a). Among these, the hypomethylated genes including *SLC16A1*, *ABHD12* and *ACOT4* were upregulated while hypermethylated genes including *SLC16A13*, *ABAT*, *ALDH4A1* and *PPP2R2B* were downregulated.

Functional classification of these genes showed that the DMRs which exhibited opposite patterns to the mRNA expression levels were found to be significantly enriched in 7 biological processes (Fig. 11b) including, plasma membrane lactate transport (*SLC16A13*), alanine, aspartate and glutamate metabolism (*ABAT, ALDH4A1*) and phosphorus metabolic processes (*GLIPR2, CTNS, ABHD12, PPP2R2B, PROM2, SERINC3* and *ACOT4*).

**Fig. 12:**
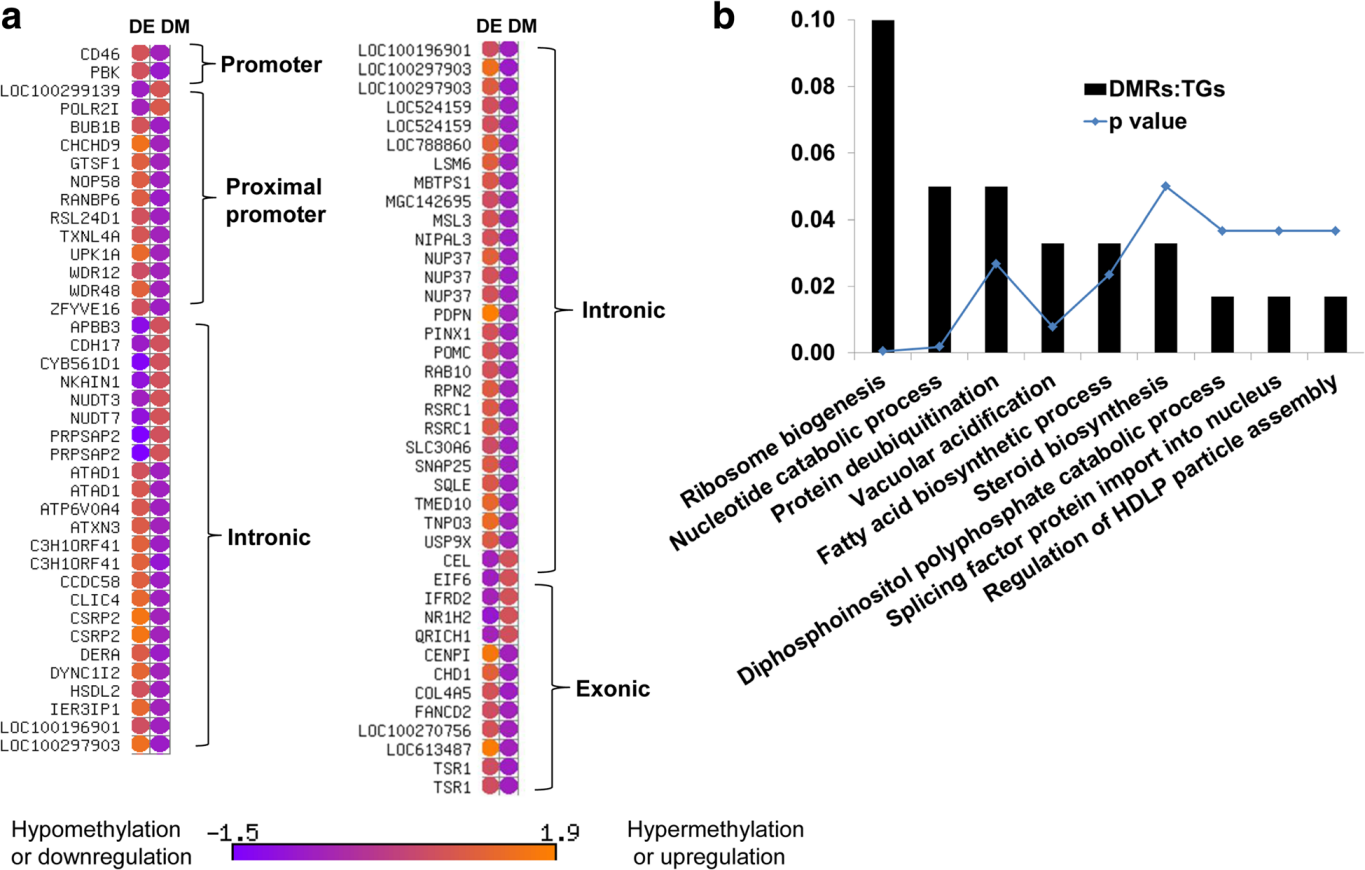
**a** The expression patterns of DMRs exclusively detected only in the 8C_Flush blastocyst group. DM; differentially methylated, DE, differentially expressed. **b** Pathways enriched by DMRs which were exclusively detected only in the 8C_Flush blastocyst group and whose methylation patterns were inversely correlated with the expression levels. DMRs:TGS, the ratio of differentially methylated genomic regions (DMRs) and the total number of genes (TGs) involved in the pathway

**Fig. 13:**
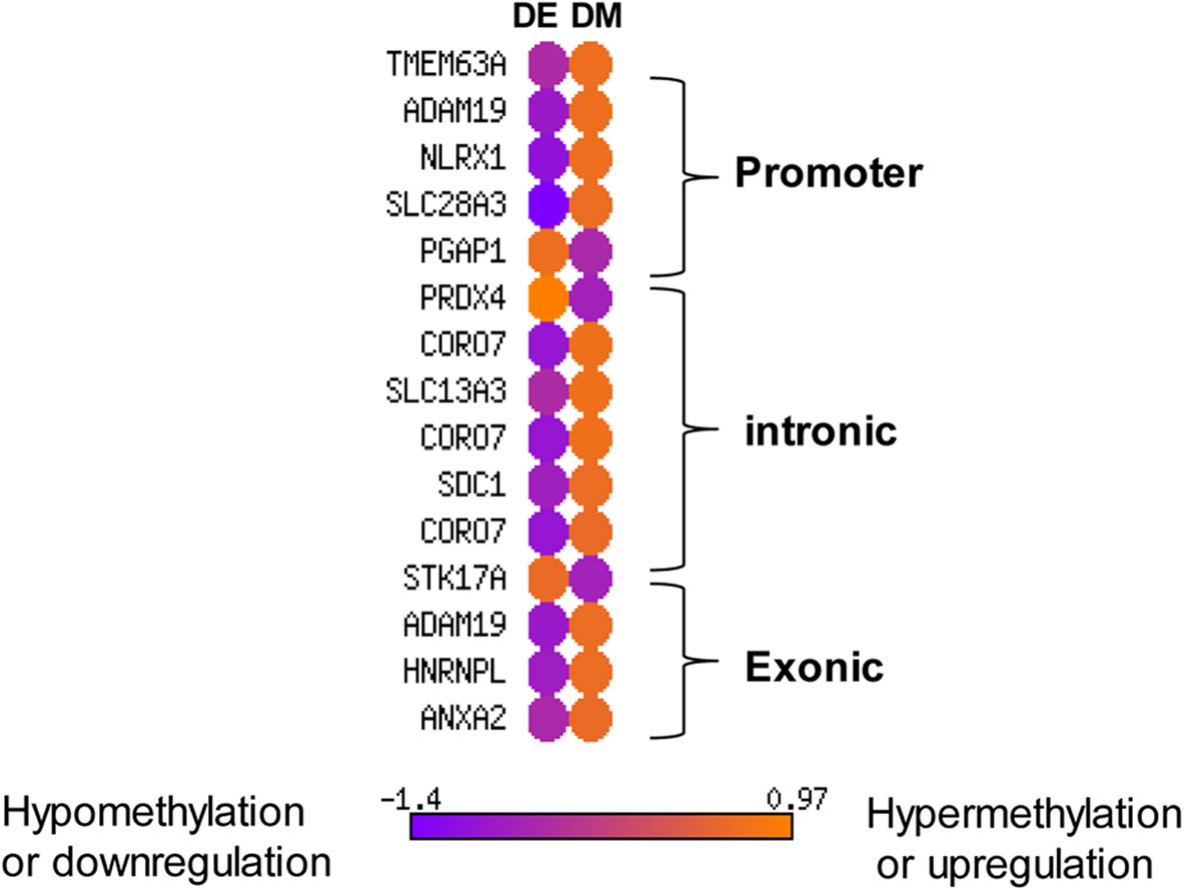
The gene expression patterns of the DMRs unique to 16C_Flush blastocyst group. DM; differentially methylated, DE, differentially expressed

In the 8C_Flush blastocyst group, a total of 67 genes including those differentially methylated in promoter regions (*PBK, TXNL4A, UPK1A* and *ZFYVE16),* in intronic region (*ATP6V0A4, NUDT3* and *TNPO3)* and exonic region (*NR1H2, TSR1* and *EIF6)* were inversely correlated with the gene expression (Fig. 12a). Functional annotation of these genes indicated that biological processes including ribosome biogenesis, protein ubiquitination, vacuolar acidification, regulation of fatty acid biosynthetic process, steroid biosynthesis and diphosphoinositol polyphosphate catabolic process were found to be altered (Fig. 12b). However, in the 16C_Flush group, only 15 differentially methylated genes including PGAP1, and PRDX4 were inversely related to the gene expression patterns (Fig. 13).

### Quantification of the DNA methylation patterns using bisulfite sequencing

**Fig. 14:**
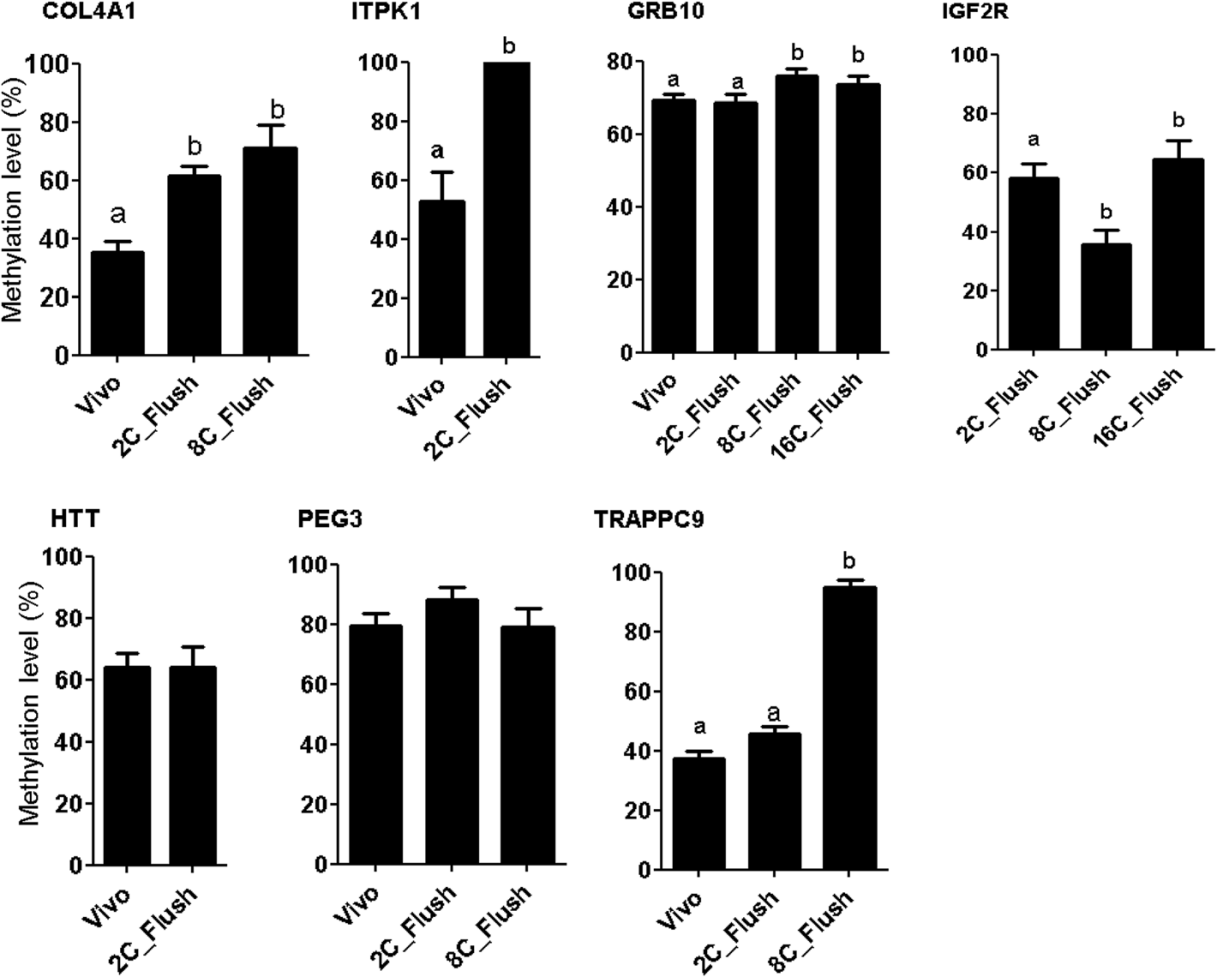
Bisulfite sequencing results of the DMRs identified by the EmbryoGENE DNA Methylation Array

The methylation patterns and the expression level of selected candidate differentially methylated imprinted (*GRB10*, *PEG3* and *IGF2R*) and non-imprinted genes (*ITPK1*, *COL4A1* and *TRAPPC9*) were analyzed using bisulfite sequencing (Fig. 14). Among genes analyzed, GRB10 which was hypermethylated in 2C_Flush, 8C_Flush and 16C_Flush blastocyst groups compared to the in vivo counterparts, and the bisulfite sequencing also confirmed hypermethylation of this gene in the 8C_Flush and 16C_Flush blastocyst groups. In addition, according to the array result *TRAPPC9*, *COL4A1* and *ITPK1* were hypermethylated in 2C_Flush, 8C_Flush and the bisulfite sequencing indicated relatively higher methylation of these genes in those samples relative to the vivo blastocysts. Therefore, results of the bisulfite sequencing were similar to the array data indicating the validity of the array analysis.

### Validation of differentially expressed genes using quantitative polymerase chain reaction (qPCR)

**Fig. 15:**
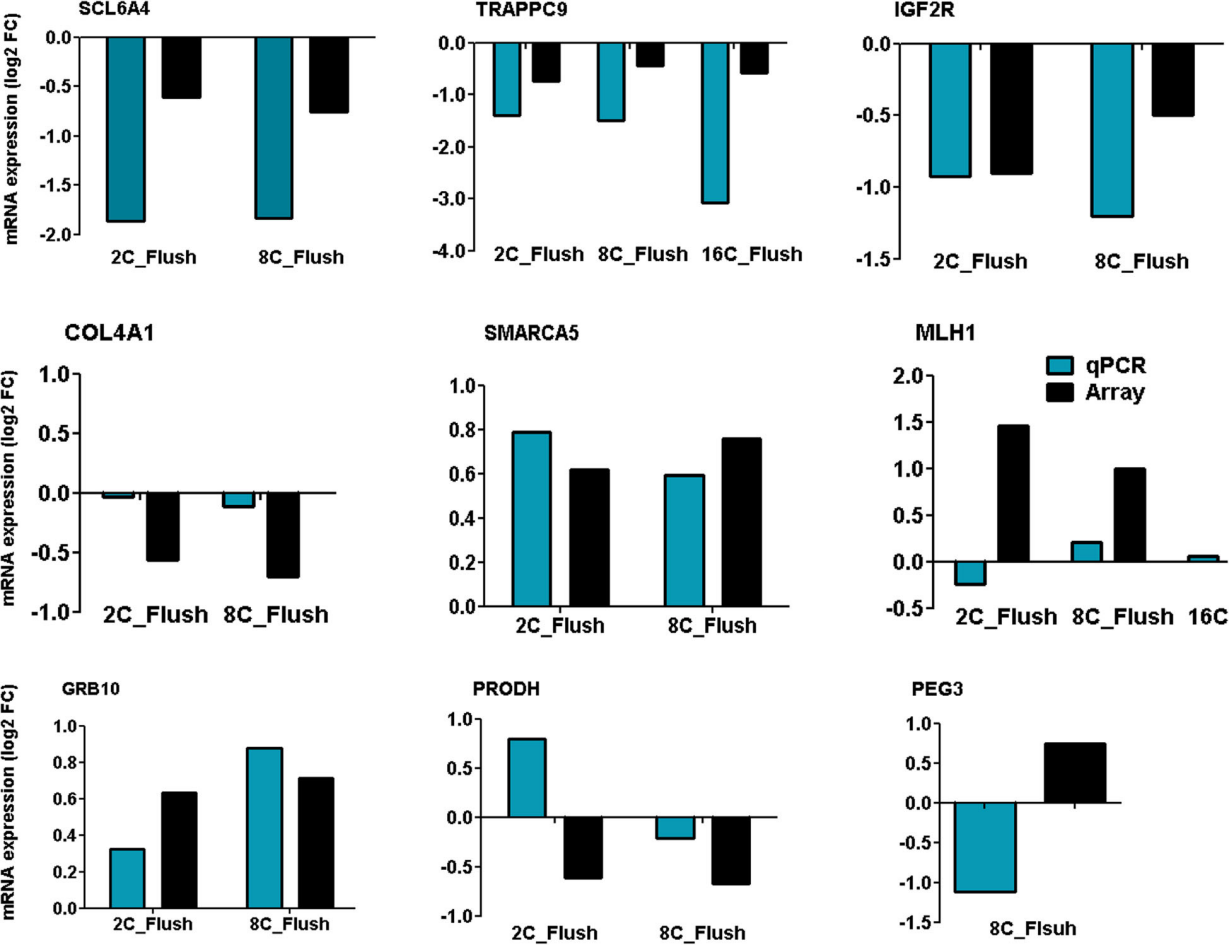
The qPCR validation results of the selected differentially expressed genes generated by EmbryoGENE transcriptome microarray

For comparative analysis of the DNA methylation and expression profile, we have selected genes which showed a difference of log_2_ fold change ≥0.56, or log_2_ fold change ≤ − 0.56 with *p* value < 0.05 in the previous dataset. Therefore, the expression patterns of nine candidate differentially expressed genes were validated using the qPCR using the total RNA isolated from independent samples. The results indicated that of 15 out 18 comparisons were found to be in line to the array data (Fig. 15). Furthermore, the qPCR data indicated that the expression levels of the hypermethylated genes, namely *TRAPPC9*, *COL4A1*, *PEG3*, *IGFR2* in 2C_Flush and 8C_Flush (Fig. 14) were found to be downregulated but the expression level of *GRB10* was upregulated (Fig. 13).

## Discussion

In vitro embryo production is one of one of the most important innovations and one of the sustainable strategies to meet the global milk and meat demands [23]. Moreover, in vitro embryo production has been one of the innovation used to foster farm animal breeding and to make a significant progress in human and veterinary medicine [24]. However, several evidences indicated that in vitro culture condition are not exactly mimicking or replacing the in vivo situation. As the result of this, only 30–40% of the in vitro produced embryos are developing to the blastocysts stage [25]. In some cases, embryos developed under in vitro culture condition can be of low quality compared to those developed in vivo and these differences are further manifested by the darker cytoplasm, low cryotolerance and lower pregnancy rate after transfer to recipients [26, 27]. Several evidences have demonstrated that these phenotypic differences are critically affected by the post- fertilization embryo culture condition which can be subsequently manifested by aberrant expression and DNA methylation pattern of developmentally related genes [11–14, 17]. However, identification of the embryonic stages which are sensitive to the environmental insults remains a challenge. Indeed, establishing an appropriate model which mimics the optimal and suboptimal embryo culture condition is indispensable to unravel the phenotypic and molecular abnormalities induced in embryo subjected to environmental challenges. Therefore, embryos developed in vivo until 2-, 8- or 16-cell stages were subjected to in vitro culture until the blastocyst stage. These embryo groups are believed to represent the developmental stages of before, during and after the onset of major embryonic genome activation, respectively. In fact, developmental data indicated that the proportion of embryos developed to the blastocyst stage, 7 days post-insemination was lower in embryonic stages subjected to in vitro culture condition before or during the onset embryonic genome activation compared to those exposed after the onset of embryonic genome activation. Interestingly, the blastocysts from these embryo groups exhibited a higher DNA methylation dysregulation. Indeed, a detailed analysis of the DMRs indicated the presence of commonly affected genomic regions in all or at least in the two blastocyst groups irrespective of stage-specific exposure to in vitro culture condition. Accordingly, the DNA methylation patterns of several arrays of genomic loci including those involved in the focal adhesion and metabolic pathways were commonly altered in all blastocyst groups suggesting that these genomic regions are sensitive to the environmental insults at any stages of preimplantation embryonic development. For instance, the imprinted genes such as *PEG3*, *IGF2R*, *SNRRP* and *GRB10 (*Table 2) were aberrantly methylated in all blastocysts and independent bisulfite sequencing. We also observed differences in the DNA methylation level of *PEG3, IGF2R and GRB10* genes between the blastocyst groups. Culture condition induced aberrant methylations of the imprinted genes in preimplantation embryos have been highlighted in many instances. For instance, culture condition-induced aberrant DNA methylation pattern of *SNRRP* in bovine embryos [28] and *PEG3* gene in mouse placenta [29] have been indicated. Moreover, aberrant DNA methylation pattern of *PEG3, IGF2R* and *GRB10* in children conceived by IVF/ICSI [30] also described the long term effect of suboptimal culture condition on the DNA methylation pattern of imprinted genes. This, could in turn, reduce embryonic developmental competence and/or result in fetal abnormalities. For instance, aberrant methylation of *IGF2R* could cause large offspring syndrome in sheep [31] and functional loss of *GRB10*, during embryonic development was also resulted in overgrowth of both the embryo and placenta in human [32].

**Table 2:** List of commonly differentially methylated imprinted genes and DNMTs

Gene symbol	2C_Flush	8C_Flush	16C_Flush	Genomic region
PEG3	▲	▲	▲	Gene body
GRB10	▲	▲	▲	Gene body
IGF2R	▲	▲	▲	Gene body
DNMT3B	▲	▲	-	Gene body
DNMT1	▼	▲	▲	Gene body
ASB4	▲	▼	▼	Gene body
SFMBT2	▲	▼	▼	Gene body

▲ Hypermethylated, ▼ Hypomethylated

In addition to imprinted genes, cluster of genes such as the solute carriers and the zinc finger proteins were commonly differentially methylated in all blastocyst groups. The solute carriers are believed to be essential for transporting cellular materials across the cell membrane and altered DNA methylation of the solute carrier genes in the developing embryos could negatively affect the proper functioning of protein transportation across the cell membrane.

In addition to these, aberrant DNA methylation of the zinc-finger proteins such as *ZNF234, ZNF618, ZNF688* and *ZNF862* are interesting as the zinc proteins are found to further regulate the DNA methylation patterns of some other genes by targeting the M.CviPI (GC methylation) or M.SssI (CG methylation) sites [33]. On the other hand, extracellular-matrix related genes including *COL4A1* and *COL1A2* were also differentially methylated in all blastocyst groups of which the *COL4A1* gene was hypermethylated in all blastocyst groups and its expression pattern was downregulated. The DNA methylation and expression patterns of *COL4A1* were further confirmed by bisulfite sequencing and qPCR, respectively using independent blastocyst samples. Accordingly, COL4A1 gene was hypermethylated, but its expression was downregulated in blastocyst obtained from 2- and 8-cell flush embryos compared to completely in vivo developed blastocysts. The functional relevance of these gene in bovine embryo development is barely understood, but one study showed that functional loss of *COL4A1* and *COL4A2* resulted in embryonic lethality in mouse embryo due to impaired basement membrane stability [34]. Moreover aberrant DNA methylation pattern of COL4A1 gene in the gene body was associated with cancer [35].

Apart from the DNA marks altered in all blastocyst groups; we have also analyzed the DNA methylation marks, which are specific to each of the blastocyst group. Unlike the blastocysts of the 8-cell flush, the blastocysts of the 2- and 16-cell flush groups were associated with more hypermethylated genomic loci than the hypomethylated ones, suggesting that exposure of embryos before or after embryonic genome activation could induce DNA methylation, while exposing at the time of genome activation could foster demethylation of the resulting blastocysts. Indeed, the interdependence between the onset of genome activation and DNA methylation patterns during mammalian embryogenesis is barely understood. In fact, the genome DNA undergoes multiples epigenetic modifications immediately after fertilization which may in turn affect the gene expression patterns that could be established during and after embryonic genome activation. For instance, active and passive demethylation of the pronuclei occurs during mouse, bovine and human embryonic development, but the timing of re-methylation is different depending on the species [8, 36, 37]. In mice and humans, massive de novo methylation occurs at the blastocyst stage [8, 38], whereas in bovine, the major embryo genome activation is believed to occur between the 8- and 16-cell stage [39] although the bovine embryo has been found to be transcriptionally competent at the 2-cell stage [40]. Conceptually, for genes to be expressed, the DNA sequence must be accessible for the transcriptional machinery and any blockage of the transcription machinery could potentially shutdown the gene expression [41]. Among these, hypomethylation (removal of the DNA methylation marks) around the promoter region is believed to increase the transcriptional activity of the genes [42–44]. During normal embryo development, the fertilized egg undergoes multiple epigenetic modifications, which subsequently affect the resulting gene expression profiles. In mice, while the paternal pronucleus undergoes active demethylation, the maternal genome passively demethylated after the 2-cell stage [45]. However, in bovine, the beginning of de novo methylation appears to overlap the genome activation occurring at the same cell stage. Nevertheless, whether this pattern remains similar while embryos are exposed to the suboptimal environmental condition is not clear. However, the study in rabbit embryo showed that DNA demethylation in in vitro produced embryos occurs between the 2- and 8-cell stages while in in vivo ones, demethylation occurs between 4- and 16-cell stages and remains constant from 16-cell until the morula stage suggesting that demethylation and remethylation of the genome occurs faster in in vitro embryos than the in vivo ones. Similarly, the study by Desmet [28] also indicated differences in the DNA methylation changes when early stage embryos are exposed to suboptimal culture suggesting that early-stage embryos could be more susceptible to adverse culture condition.

In addition to the holistic approaches, we have also looked into the developmental relevance of uniquely differentially methylated individual genes in each blastocyst group. For instance, some of the Krüppel-like transcription factors (KLFs) were differentially methylated in blastocysts of the 2-cell flush group. The KLFs are believed to be evolutionarily conserved and potentially involved in several cellular activities such as proliferation, differentiation and cellular development [46]. These families of genes are consisting of three Krüppel-like zinc fingers which bind to CACCC elements and GC-rich regions of DNA for mediating the gene transcription [47]. Among the KLFs, KIF4 was among the differentially methylated KLFs in blastocysts of the 2-cell flush. KLF4 is involved in cell proliferation, apoptosis and invasion [48] and believed to be negatively regulating the expression of endodermal markers which may be involved in the transformation of pluripotent stem cells to endoderm differentiation [49]. In addition, KLF4 regulates the NANOG and OCT3/4 transcription during embryonic stem cell self-renewal and subsequently block the embryonic stem cell differentiation [50, 51]. Therefore, developmental retardation of the 2-cell flush may partly be induced by aberrant DNA methylation pattern of the KLFs.

Overall, in this study we have identified several DMRs which are sensitive to culture condition at any stage of embryonic development. Hypermethylated or hypomethylated genomic loci are generally believed to play a role in regulating cellular development and function by inhibiting or inducing the gene expression. Therefore, in this study, apart from exploring the molecular pathways enriched by the DMRs, we also investigated the effects of those DMRs on the gene expression patterns. Accordingly, we have detected about 2–9% of annotated DMRs of in the promoter or gene body regions were inversely correlated with the corresponding gene expression (Figs. 5, 7, 11, 12 & 13). Interestingly, these genes were found to be involved in key pathways that are believed to be essential for embryogenesis such as fatty acid biosynthetic process, steroid biosynthesis, energy metabolism and JAK-STAT cascade pathways. Indeed, although the proportion of DMRs that inversely correlated with the gene expression seems to be lower while considering the total number of genomic loci differentially methylated, it should be noted that there were several DMRs whose expression was positively correlated with gene expression (data not shown). On the other hand, the DMRs whose mRNA was not changed does not necessarily imply that their methylation pattern is not essential for mRNA expression, rather these DMRs could be involved in regulating the embryonic development by regulating the downstream genes or by fine-tuning the expression pattern of other gene regulatory agents such as miRNAs.

## Conclusion

This study identified culture-induced stage-specific and non-stage-specific aberrant DNA methylation patterns of paternally or maternally imprinted genes and several arrays of genomic regions in in vivo originated bovine embryos subjected to the in vitro culture in stage specific manner. Thus, embryos exposed to in vitro culture condition before the onset or during embryonic genome activation are more sensitive to the DNA methylation marks changes that could be further displayed in the resulting blastocysts compared to those exposed to in vitro culture condition after embryonic genome activation. Thus, while exposure of in vivo embryos to in vitro culture condition before embryonic genome activation favored the initiation of DNA methylation, subjecting of in vivo embryos to the in vitro culture at the time of genome activation increased the hypomethylated genomic loci in the resulting blastocysts. Nevertheless, a detailed study is required to understand whether these altered DNA methylation patterns induced in the embryo by suboptimal culture stimuli are initiated as means of coping mechanisms of the embryo or an indicator of abnormal embryo development. Moreover, future studies could further investigate whether or not the suboptimal culture-induced epigenetic modifications occurred during bovine preimplantation embryo at the blastocysts stage could have a long-lasting consequence on the offspring epigenome.

## Additional files

Additional file 1: **Table S1.** The list of primers used to validate DMRs in blastocysts of different groups using bisulfite sequencing. (DOCX 15 kb)

Additional file 2: **Table S2.** The list of primers used for validation of the differentially expressed genes using qPCR. (DOC 33 kb)

Additional file 3: **Figure S1.** The order of chromosomes based their probe density and differentially methylated probes (TIF 4242 kb)

Additional file 4: **Figure S2.** The circos plot representing the overall methylation levels in the in different blastocyst groups**.** Positive and negative fold-changes represent the level of hypermethylation and hypomethylation. (PDF 19412 kb)

Additional file 5: **Figure S3.** Genomic distribution of exclusively differentially methylated regions in the 2C_Flush, 8C_Flush or 16C_Flush blastocyst group. (PDF 47 kb)

## Abbreviations

16C_Flush: blastocysts developed in vitro from embryos flushed at 16-cell stage
2C_Flush: blastocysts developed in vitro from embryos flushed at 2-cell stage
8C_Flush: blastocysts developed in vitro from embryos flushed at 8-cell stage
CpG: Cytosine phosphate guanine
DE: Differentially expressed
DM: Differentially methylated
DMRs: Differentially methylated genomic regions
EDMA: Embryogene DNA methylation array
EGA: Embryonic genome activation
EGFR: Epidermal growth factor receptor
FDR: False discovery rate
ICSI: Intracytoplasmic sperm injection
IVF: In vitro fertilization
qPCR: Quantitative real time polymerase chain reaction
