# Supplementary material for: Genome-wide DNA methylation patterns of bovine blastocysts derived from in vivo embryos subjected to in vitro culture before, during or after embryonic genome activation
Source: BMC Genomics. 2018 Jun 1;19:424. doi: 10.1186/s12864-018-4826-3 (PMC5984773; doi:10.1186/s12864-018-4826-3)
Supplement: Supplementary file 1 — Table S1. The list of primers used to validate DMRs in blastocysts of different groups using bisulfite sequencing. (DOCX 15 kb) [file 12864_2018_4826_MOESM1_ESM.docx]

Table S1: The list of primers used to validate DMRs in blastocysts of different groups using bisulfite sequencing

| EDMA_ID | Associated gene | Primers (5’—3’) | bp |
| --- | --- | --- | --- |
| MET_18_17537 | *PEG3* | F: TGGTTGTTGGATATTATAAAGGATG  R: CAAATAAACTAAAATCCCTCCTCAC | 209 |
| MET_04_00443 | *GRB10* | F: TTGGGGTTATAGAGTTAGGGAGTG  R: CTCACAATTCAACCAAAATAAAATC | 172 |
| MET_09_09328 | *IGF2R* | F: GGGTTAGTTTAGTTTTTTTTGTATTTTA  R: CAAAATAATCACTTACCTCCTAAACAC | 228 |
| MET_06_09550 | HTT | F: TGTGGTTTAGGATGTTTTTTT  R: TCCTTTATCTACAACCTCAATC | 230 |
| MET_21_08314 | *ITPK1* | F: TGTTAGGGGTTGGTAGGATGA  R: CCCCCTCTTCCTTTCCTAAATA | 187 |
| MET_12_09573 | *COL4A1* | F: AGATTGAATTGAATTGAATTGT  R: AAAATCAAAAAACCAAAATAAA | 222 |

bp= PCR product length in base pairs, EDMA_ID refers to the identification of each EDMA probe.
