# Supplementary material for: Genome-wide DNA methylation patterns of bovine blastocysts derived from in vivo embryos subjected to in vitro culture before, during or after embryonic genome activation
Source: BMC Genomics. 2018 Jun 1;19:424. doi: 10.1186/s12864-018-4826-3 (PMC5984773; doi:10.1186/s12864-018-4826-3)
Supplement: Supplementary file 2 — Table S2. The list of primers used for validation of the differentially expressed genes using qPCR. (DOC 33 kb) [file 12864_2018_4826_MOESM2_ESM.doc]

| Accession number | Genes | Primer sequence (5’-3)’ | Size (base pairs) |
| --- | --- | --- | --- |
| NM_001034034 | GAPDH | F: ACCCAGAAGACTGTGGATGG  R: ACGCCTGCTTCACCACCTT | 247 |
| NM_001166511 | COL4A1 | F: TCTGGATCGGCTACTCCTTT  R: AACATCTCGCTCCGCTCTAT | 196 |
| NM_001002887.2 | PEG3 | F: CCAAGGAAAGAGAGCACAGA  R: GCACTTCTCTTTGACCTTGC | 235 |
| NM_001075185.1 | PRODH | F: CACGCTGATGAAGATGACCT  R: GAACTGCTTTTCCCTCTTACTG | 226 |
| NM_174609.2 | SLC6A4 | F: ACTGCACCAACTACTTCTCC  R: GATGTTTTGACGCCTTTCCA | 208 |
| NM_001192586.1 | GRB10 | F: ACAAGCAAAGTGGTGGAGAT  R: CCATCTGTTCTGGGAAGAAAT | 268 |
| NM_001105416.1 | SMARCA5 | F: TGCACATTTCATTCAGCCTG  R: TGGAGAGTCTTCAAACCGAG | 211 |
| NM_001075994.2 | MLH1 | F: GAGGACGATGCAACAATGAG  R: AGGTTGATGATCCTTCTCCG | 173 |
| XM_005215216.2 | TRAPPC9 | F: AGACATTACAAGAAGCGGTGC  R: AGTGATAGATGACAGAGGCCG | 151 |

Table S2: The list of primers used for validation of the differentially expressed genes using qPCR
