# Supplementary figures and images for: Genome-wide DNA methylation patterns of bovine blastocysts derived from in vivo embryos subjected to in vitro culture before, during or after embryonic genome activation
Source: BMC Genomics. 2018 Jun 1;19:424. doi: 10.1186/s12864-018-4826-3 (PMC5984773; doi:10.1186/s12864-018-4826-3)

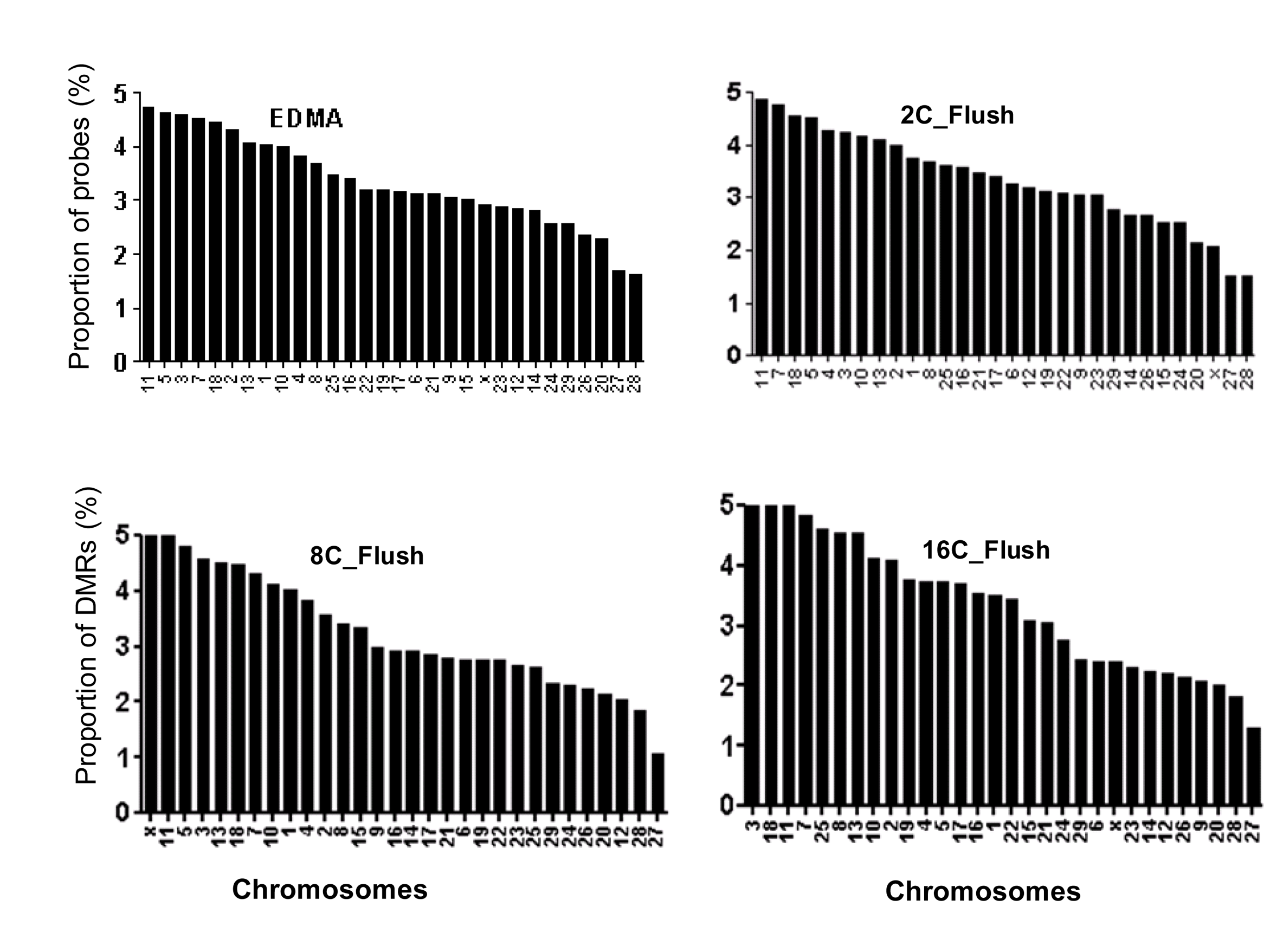

Supplement: Supplementary file 3 — Figure S1. The order of chromosomes based their probe density and differentially methylated probes (TIF 4242 kb) [file 12864_2018_4826_MOESM3_ESM.tif]

2C\_Flush

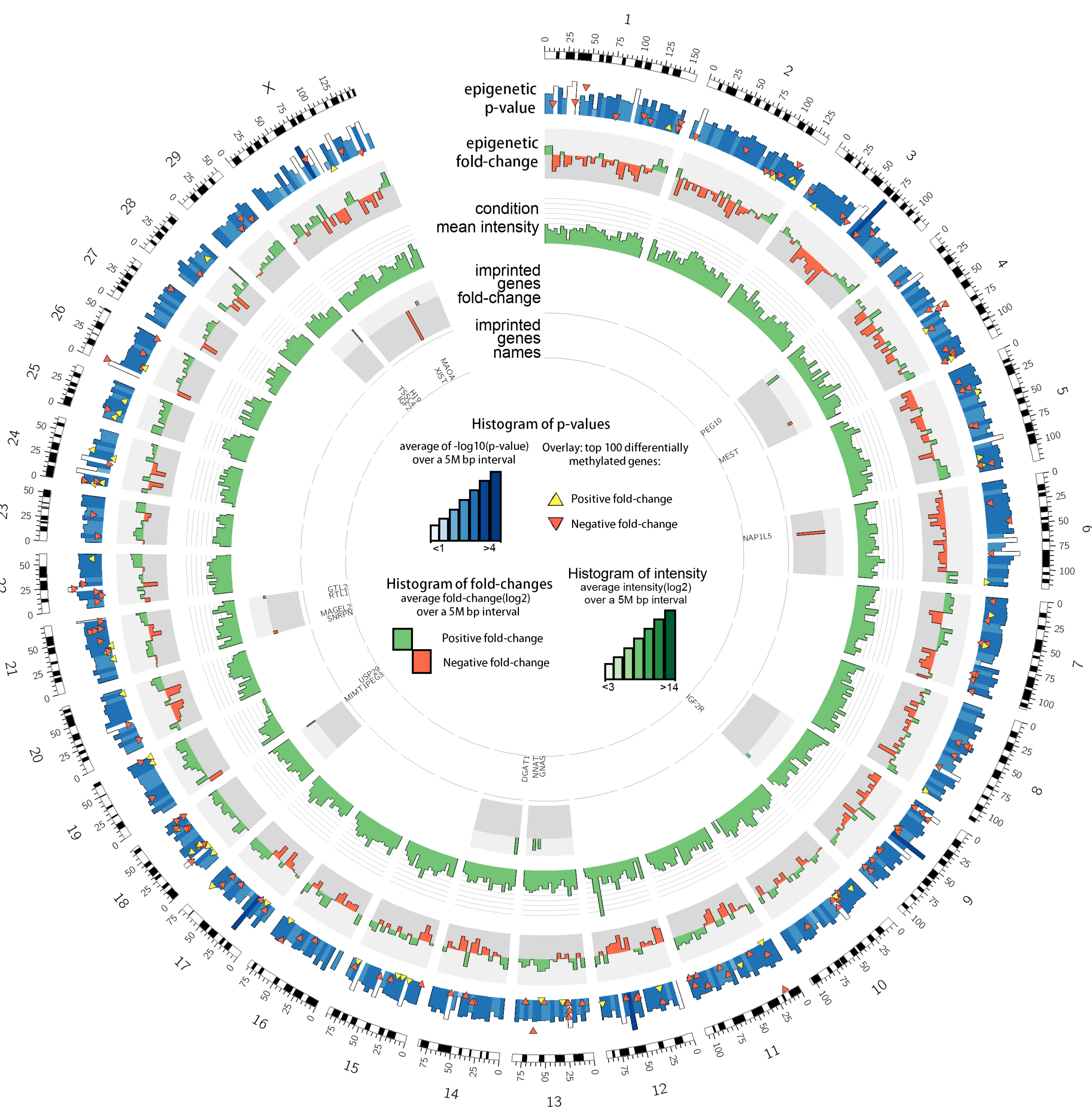

8C\_Flush

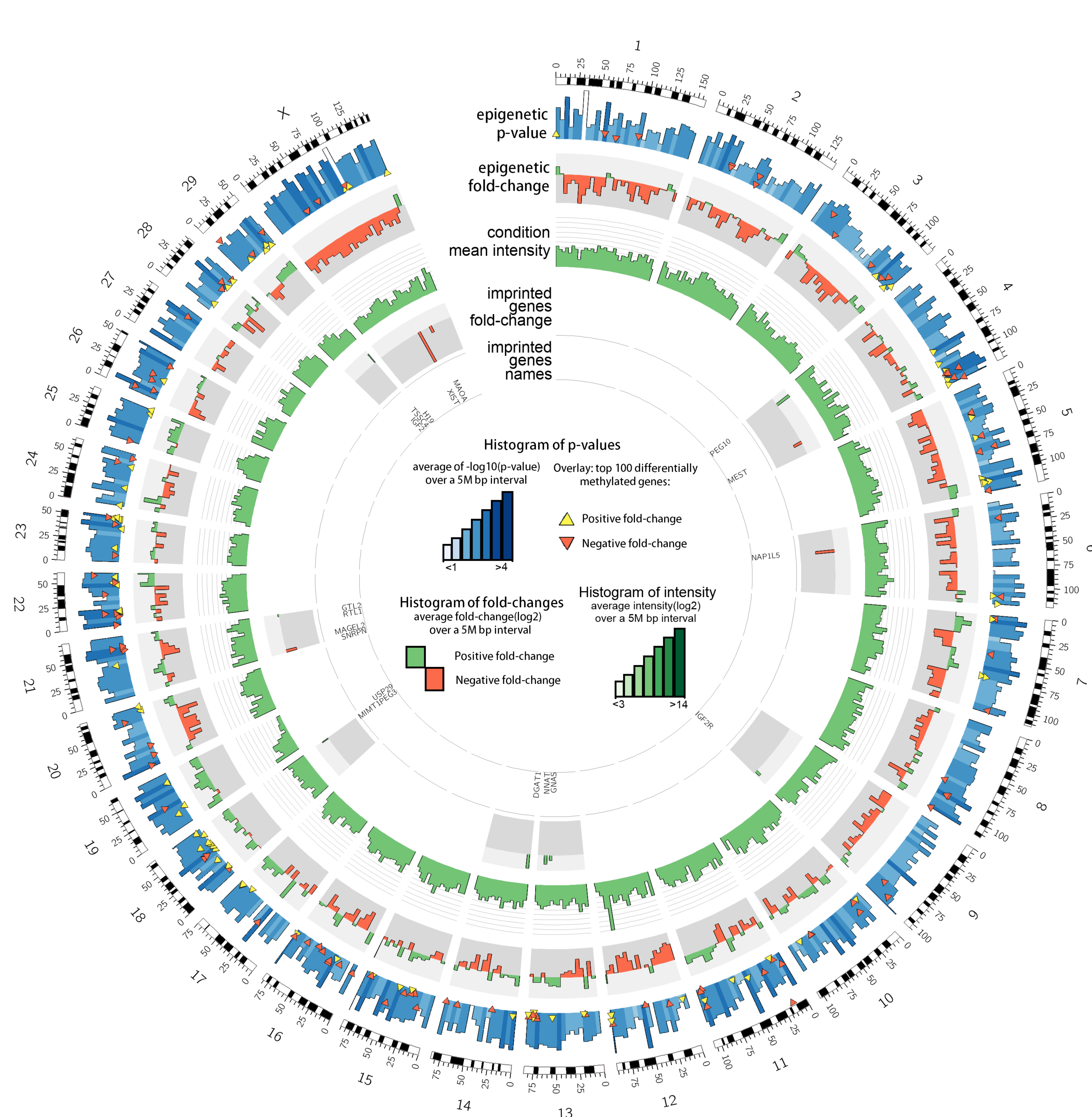

16C\_Flush

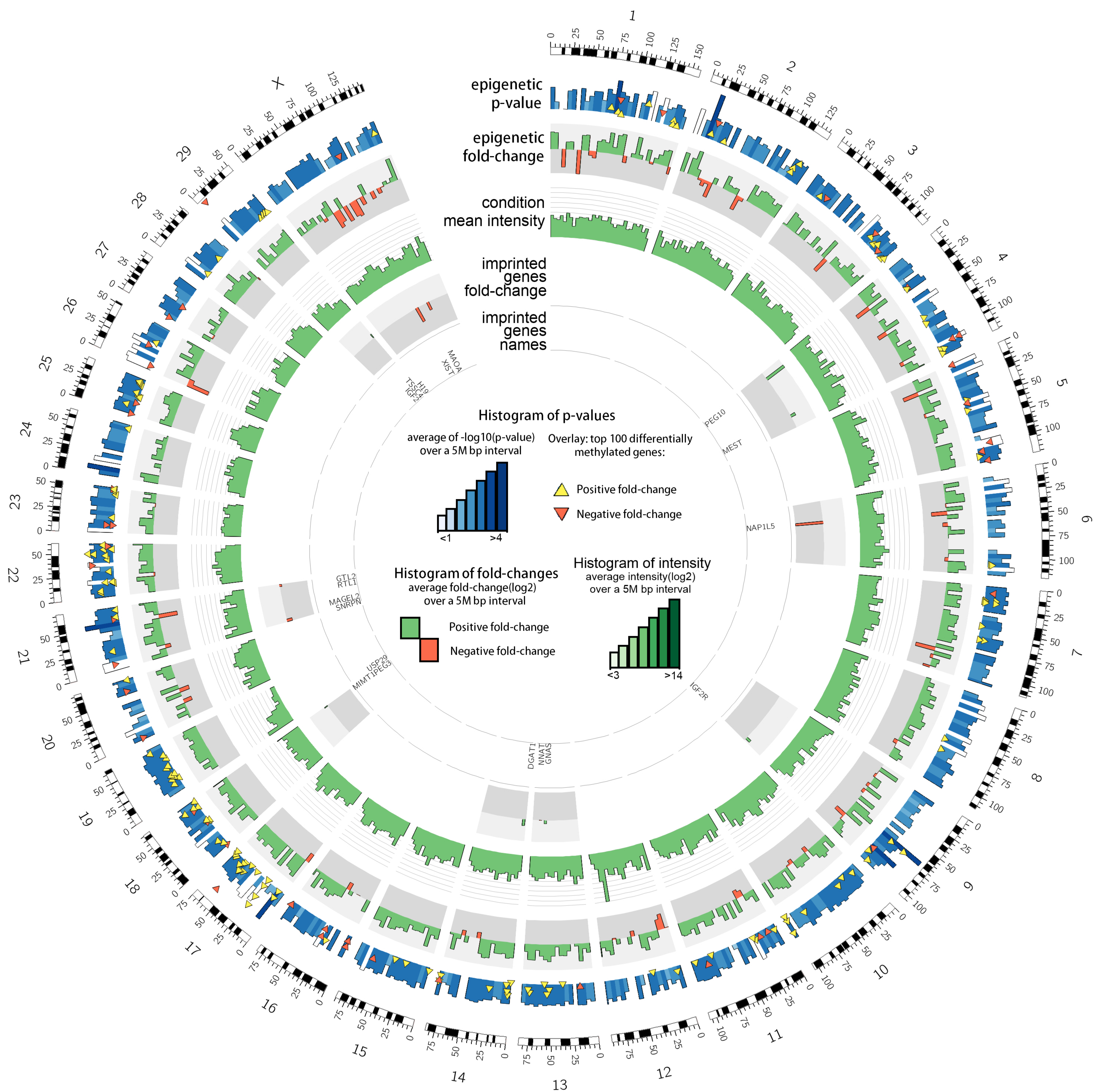

Supplement: Supplementary file 4 — Figure S2. The circos plot representing the overall methylation levels in the in different blastocyst groups. Positive and negative fold-changes represent the level of hypermethylation and hypomethylation. (PDF 19412 kb) [file 12864_2018_4826_MOESM4_ESM.pdf]

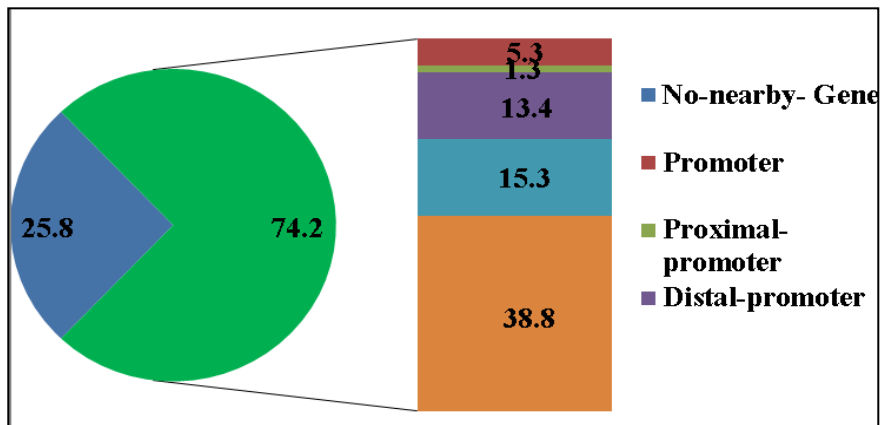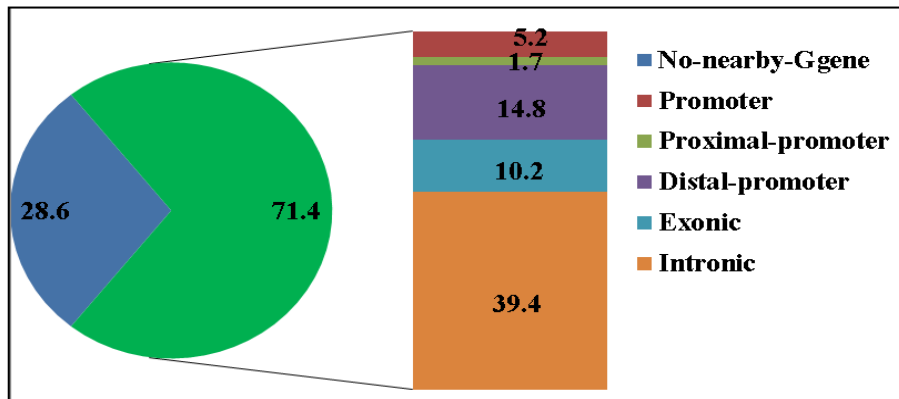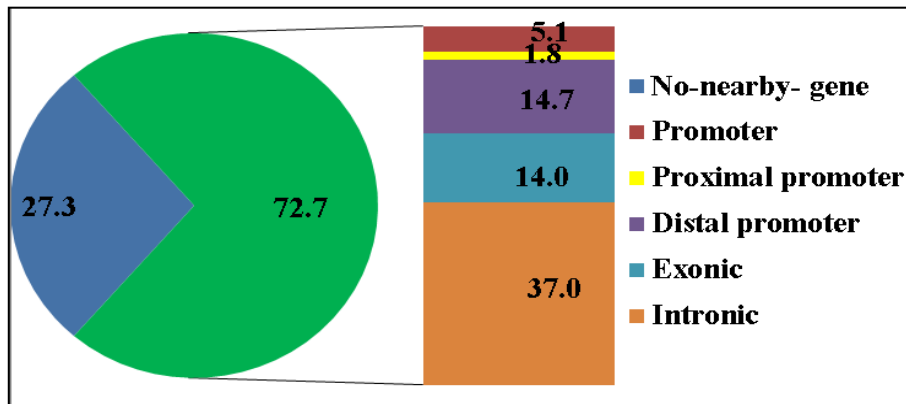

Supplement: Supplementary file 5 — Figure S3. Genomic distribution of exclusively differentially methylated regions in the 2C_Flush, 8C_Flush or 16C_Flush blastocyst group. (PDF 47 kb) [file 12864_2018_4826_MOESM5_ESM.pdf]
